# NAT10 promotes cisplatin resistance and immune escape by increasing the expression of DUSP1 and PD-L1 in gastric cancer

**DOI:** 10.1038/s41420-026-03107-w

**Published:** 2026-04-10

**Authors:** Lilin Qian, Wenrong Gao, Xinyi Wang, Shuqi Cui, Xiaoqi Han, Xia Xu, Jihui Jia, Zhifang Liu

**Affiliations:** 1 Department of Biochemistry and Molecular Biology, Key Laboratory for Experimental Teratology of Chinese Ministry of Education, School of Basic Medical Sciences, Cheeloo College of Medicine, Shandong University, Jinan, PR China; 2 Department of Microbiology, Key Laboratory for Experimental Teratology of Chinese Ministry of Education, School of Basic Medical Sciences, Cheeloo College of Medicine, Shandong University, Jinan, PR China

**Keywords:** Gastric cancer, Oncogenes

## Abstract

Developing resistance to chemotherapy drugs and evading the killing effect of the immune system are the main obstacles in the clinical treatment of gastric cancer. However, the potential mechanism remains poorly understood. N-acetyltransferase 10 (NAT10) catalyzes the N4-acetylcytidine (ac4C) modification of mRNA and is associated with tumor occurrence, development and chemotherapy resistance. Here, we observed that elevated NAT10 levels promote cisplatin chemoresistance in gastric cancer cells. On the contrary, knockdown of NAT10 enhances the sensitivity of cisplatin-resistant gastric cancer cells to cisplatin, both in vitro and in vivo. Mechanistically, NAT10 binds to DUSP1 mRNA and catalyzes its ac4C modification at positions C327, C330, and C331 within the coding sequence (CDS) region, thereby enhancing the stability of DUSP1 mRNA and increasing the abundance of DUSP1 protein. Furthermore, NAT10 mediates resistance to cisplatin-induced apoptosis through DUSP1 via the JNK and ERK signaling pathways. Additionally, NAT10 can upregulate PD-L1 expression via FOSB. The combination of a NAT10 inhibitor and an anti-PD-1 antibody synergistically enhances the antitumor efficacy against cisplatin- resistant gastric cancer cells in murine models. Taken together, these findings offer novel insights into the role and mechanism of NAT10 in the crosstalk between cisplatin chemoresistance and immunosuppression in gastric cancer. NAT10 thus holds promise as a highly attractive target, with the potential to synergize with PD-1-based immunotherapy to reverse cisplatin resistance in gastric cancer.

## Introduction

Gastric cancer ranks among the most prevalent malignant tumors globally, boasting the fifth-highest incidence and mortality rates worldwide [1, 2]. An important reason for the persistently high mortality rate among gastric cancer patients is that they are often diagnosed at an advanced stage and have a poor response to the existing treatment methods [3, 4]. Cisplatin-based chemotherapy is the primary treatment for advanced gastric cancer [5]. However, acquired resistance to cisplatin is the most critical challenge affecting clinical efficacy [6–9]. In addition, evidence shows that tumor chemotherapy often increases the resistance of tumor cells to antitumor immunity [10, 11]. Therefore, further exploring the mechanisms of cisplatin resistance in gastric cancer, and then precisely formulating strategies to enhance the sensitivity of gastric cancer cells to cisplatin, is of utmost critical for improving the prognosis and elevating the survival period of patients with advanced gastric cancer.

Evidence suggests that epigenetic modifications play a significant role in drug resistance [12, 13]. Epigenetics refers to “heritable” changes in cellular functions and phenotypes that occur without any alterations to the DNA sequence [14]. It encompasses epitranscriptomics and epiproteomics. The former focuses on post- transcriptional RNA modifications, while the latter delves into post-translational modifications of proteins [14, 15]. Recent studies have found that the post-transcriptional RNA modifications exert pivotal regulatory functions in gene expression and impact multiple cellular functions [16]. N4-acetylcytidine (ac4C) modification of RNAs is one of the important forms of RNA posttranscriptional modification, playing a crucial role in regulating the stability and translation process of mRNA [17]. The ac4C modification of mRNA is catalyzed by the N-acetyltransferase 10 (NAT10), which is the only known ac4C “writer” protein to date [18]. As a member of the GCN5-related N-acetyltransferase (GNAT) family, NAT10 displays dual functionality, encompassing both acetyltransferase activity and RNA-binding capacity [19]. The aberrant expression of NAT10 has been found in a variety of tumors, and is associated with cancer progression. For example, NAT10 has been found to promote cancer progression in bladder cancer [20], prostate cancer [21], colorectal cancer [22], gastric cancer [23, 24], hepatocellular carcinoma [25] and hepatoblastoma [26] and to drive glycolysis addiction in gastric cancer [27]. Furthermore, NAT10 can induce cisplatin resistance by promoting DNA damage repair in bladder cancer [28]. In hepatocellular carcinoma, it induces lenvatinib resistance through acetylating HSP90AA1 [29]. Additionally, NAT10 promotes immunosuppression in cervical cancer [30] and triple-negative breast cancer [31]. However, the role and mechanisms of ac4C modification and NAT10 in the crosstalk between chemoresistance and chemotherapy-mediated immune evasion in gastric cancer remain unreported.

In the present study, we found that overexpression of NAT10 reduced the sensitivity of gastric cancer cells to cisplatin, whereas its knockdown significantly enhanced the sensitivity of cisplatin-resistant gastric cancer cells to cisplatin. Subsequently, we demonstrated that NAT10 induces ac4C modification of dual specificity phosphatase 1 (DUSP1) mRNA, maintains the stability of DUSP1 mRNA, and thereby increases the abundance of DUSP1 protein. NAT10 induces cisplatin resistance through DUSP1 via the JNK and ERK signaling pathways. Additionally, NAT10 upregulates the expression of programmed cell death ligand 1 (PD-L1) through the FosB proto-oncogene (FOSB). Inhibition of NAT10 significantly decreases PD-L1 abundance in gastric cancer (GC) cells and enhances the antitumor efficacy of anti-PD-1 antibodies in mouse xenograft models in vivo. Therefore, targeting NAT10 may represent a promising therapeutic strategy to concurrently overcome cisplatin resistance and alleviate immunosuppression in treatment-resistant gastric cancer cells.

## Results

### NAT10 improves the resistance of gastric cancer cells to cisplatin depending on its acetyltransferase activity

**Fig. 1:**
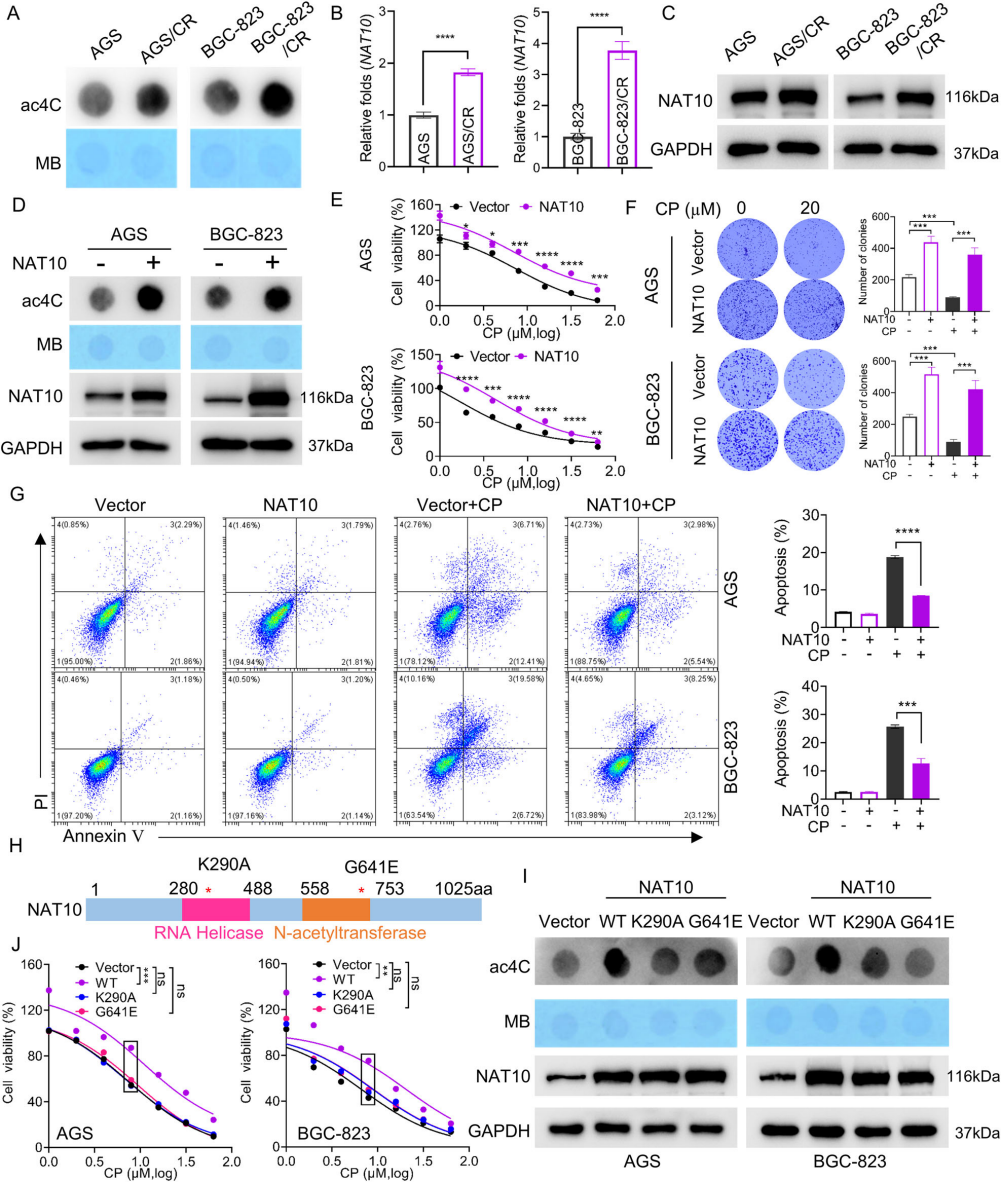
NAT10 improves the resistance of gastric cancer cells to cisplatin depending on its acetyltransferase activity. **A** Total RNA ac4C modification levels in parental cells and cisplatin-resistant cells were detected. MB, methylene blue (as internal reference). **B**, **C** Expression levels of NAT10 were examined by RT-qPCR (**B**) and Western blot (**C**) in parental cells and cisplatin-resistant cells. **D** Total RNA ac4C modification levels and NAT10 expression levels were detected via RNA dot blot and western blot assays in gastric cancer cells after NAT10 overexpression. **E**–**G** Cell viability, clonogenic capacity and cell apoptosis were evaluated using CCK-8 assays (**E**) colony formation assays (**F**) and flow cytometry (**G**) in gastric cancer cells with NAT10 overexpression and subsequent treatment with cisplatin. **H** Schematic of NAT10 functional domains and point mutations. **I** The total RNA ac4C modification levels and NAT10 expression levels were measured using RNA dot blot assays in AGS and BGC-823 cells that were transfected with an empty vector, a wild-type NAT10 expression vector, or the K290A or G641E NAT10 mutant. **J** Cell viability was determined in AGS and BGC-823 cells with the above-mentioned transfection.Statistical significance was determined by a two-tailed unpaired Student’s t - test. Error bars show the SDs from three independent experiments. *p < 0.05, **p < 0.01, *** *p* < 0.001, **** *p* < 0.0001 and ns, no significance.

In order to clarify the mechanism of platinum-resistance in gastric cancer, we established cisplatin-resistant gastric cancer cell lines (AGS/CR and BGC-823/CR) by continuously adding cisplatin into AGS and BGC-823 cells. The CCK-8 assay revealed that the half-maximal inhibitory concentration (IC_50_) values in AGS/CR and BGC-823/CR cells were significantly higher than those in the parental cells (Sup. Fig.1). Given that ac4C modification is an important post-transcriptional modification of mRNA and participates in numerous physiological and pathological processes by maintaining mRNA stability [19], we detected global ac4C modification levels in cisplatin-resistant cells and their parental counterparts. The results revealed that ac4C modification was significantly elevated in cisplatin-resistant cells compared with parental cells (Fig. 1A). Since NAT10 is currently the only identified enzyme that catalyzes ac4C modification [19], we further detected NAT10 expression levels via RT-qPCR and Western blot in both cisplatin-resistant and parental cells. As shown in Fig. 1B, C, the mRNA and protein levels of NAT10 were significantly higher in cisplatin-resistant cells. We further confirmed that cisplatin upregulates NAT10 in gastric cancer cells by activating the NF-κB pathway and increasing p65 expression (Sup. Fig. 2A–F), which is consistent with the findings reported by Xie et al. in bladder cancer cells [28]. Subsequently, we sought to investigate whether overexpression of NAT10 enhances the resistance of gastric cancer cells to cisplatin. We transfected the NAT10 expression vector into AGS and BGC-823 cells. Western blot results confirmed the transfection efficiency, and NAT10 overexpression was found to significantly elevate the global ac4C levels in these cells (Fig. 1D). The CCK-8 and colony formation assays showed that, compared with control cells, NAT10-overexpressing cells displayed significantly increased viability and clonogenic capacity (Fig. 1E, F). The apoptosis rate induced by cisplatin was significantly decreased in gastric cancer cells with NAT10 overexpression (Fig. 1G). It has been reported that the acetyltransferase activity of NAT10 depends on the K290 residue in RNA helicase domain and G641 site in acetyltransferase domain [32, 33]. Therefore, we constructed two mutant expression vectors of NAT10 (K290A and G641E) (Fig. 1H). We transfected wild-type NAT10 (WT-NAT10) expression vector or mutant NAT10 expression vector into gastric cancer cells, respectively. The results showed that the abundance of NAT10 protein increased significantly to a similar extent in gastric cancer cells transfected with either the WT-NAT10 or the mutant NAT10 expression vector. In cells overexpressing the WT-NAT10, the ac4C level increased, whereas in cells overexpressing the mutant NAT10, the ac4C level did not change significantly (Fig. 1I). The CCK-8 assay revealed that cell viability was enhanced in cells overexpressing WT-NAT10, but not in those overexpressing mutant NAT10, following cisplatin treatment (Fig. 1J). These results indicated that NAT10 promotes cisplatin resistance in gastric cancer cells depending on its acetyltransferase activity.

### Knockdown of NAT10 alleviates chemoresistance in cisplatin-resistant gastric cancer cells

**Fig. 2:**
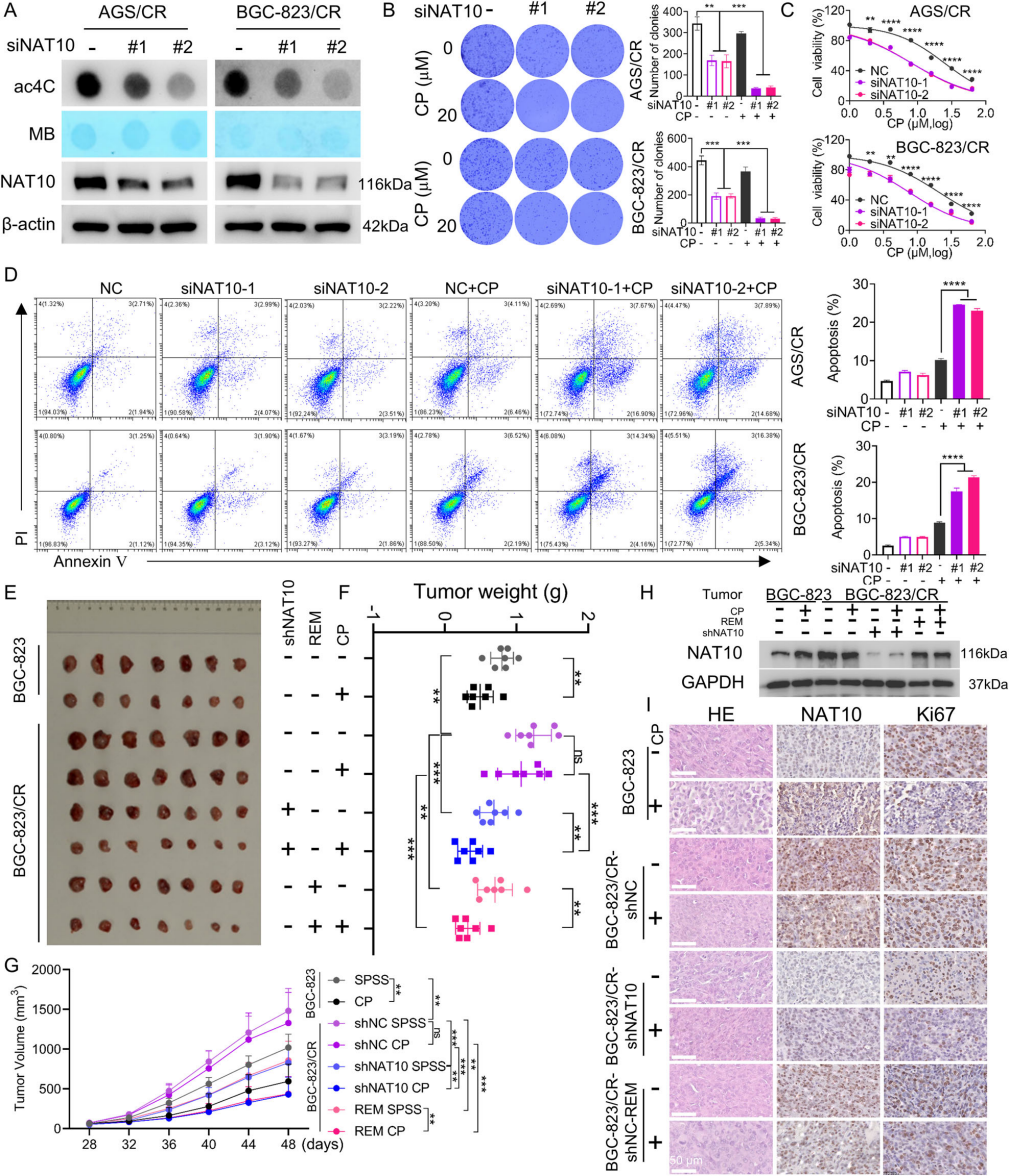
Knockdown of NAT10 alleviates chemoresistance in cisplatin-resistant gastric cancer cells. **A** Total RNA ac4C modification levels and NAT10 expression levels were detected in AGS/CR and BGC-823/CR cells transfected with siRNAs specifically targeting NAT10. **B**, **C** Colony formation assay (**B**) and CCK-8 assay (**C**) were carried out in cisplatin-resistant cells transfected with NAT10 siRNAs and then treated with PBS or cisplatin to access the colony formation ability and cell viability. **D** Flow cytometry was used to detect cell apoptosis in cisplatin-resistant cells with NAT10 knockdown and subsequent treatment with PBS or cisplatin. **E** The tumors in nude mice were surgically dissected, and the images of the tumors from the indicated treatment groups were shown. **F** Statistical analysis of the tumor weight in (**E**). **G** Tumor growth curves in the indicated groups were shown. **H** Western bolt was used to analyze the expression level of NAT10 in tumor tissues from the cohorts depicted in Fig. 2E. **I** Representative images of the HE staining results and IHC results for the expression of NAT10 and Ki67 in the above-mentioned tumors. Statistical significance was determined by a two-tailed unpaired Student’s t-test. One - way ANOVA was used to compare the means of three or more experimental groups. Error bars show the SDs from three independent experiments. *p < 0.05, **p < 0.01, *** *p* < 0.001, **** *p* < 0.0001 and ns, no significance.

To clarify the role of NAT10 in cisplatin-resistant gastric cancer cells, we transfected NAT10-specific siRNAs into AGS/CR and BGC-823/CR cells to knockdown NAT10 expression and reduce ac4C levels (Fig. 2A). Colony formation and CCK-8 assays revealed that NAT10 knockdown significantly reduced the clonogenic capacity and viability in cisplatin-treated AGS/CR and BGC-823/CR cells (Fig. 2B, C). Additionally, flow cytometry analysis showed that NAT10 knockdown increased cisplatin-induced apoptosis in these cells (Fig. 2D). We further utilized remodelin, a small-molecule inhibitor designed to specifically target NAT10, to inhibit NAT10 activity and reduce the ac4C level (Sup. Fig. 3A). CCK-8, colony formation assay and flow cytometry results showed that remodelin treatment reduced cell viability and the colony formation ability, and increased cell apoptosis rate in cisplatin-resistant gastric cancer cells, which is consistent with NAT10 interference (Sup. Fig. 3B–D).

To further confirm whether NAT10 knockdown or inhibition can enhance the sensitivity of gastric cancer cells to cisplatin treatment in vivo, we established a murine chemotherapy model. This was achieved by subcutaneously injecting cisplatin- sensitive BGC-823 cells, cisplatin-resistant BGC-823/CR control cells, and BGC-823/CR shNAT10 cells into mice, followed by cisplatin administration. The results showed that the tumor weight and volume in the BGC-823-injected group were significantly lower than those in the BGC-823/CR-injected group, indicating that BGC-823/CR cells exhibit higher malignancy than BGC-823 cells. Additionally, cisplatin treatment significantly reduced tumor weight and volume in mice injected with cisplatin-sensitive BGC-823 cells but had no effect on those injected with cisplatin-resistant BGC-823/CR control cells. As expected, compared with mice injected with BGC-823/CR control cells, cisplatin resistance was significantly reversed in mice injected with BGC-823/CR shNAT10 cells. When mice bearing BGC-823/CR control cells were treated with the NAT10 inhibitor remodelin either alone or in combination with cisplatin, remodelin not only suppressed tumor growth independently but also exerted a synergistic tumor growth-inhibitory effect when co-administered with cisplatin (Fig. 2E–G). Western blot and immunohistochemistry (IHC) assays demonstrated that cisplatin treatment induced a notable elevation of NAT10 expression in tumor tissues derived from BGC-823 cells. Additionally, the results confirmed that NAT10 expression was stably suppressed in tumor tissues originating from shNAT10-knockdown BGC-823/CR cells, thereby validating the efficacy of the shRNA-mediated knockdown strategy (Fig. 2H, I). Furthermore, IHC staining results revealed that relative to the control group, Ki67 expression was reduced in tumors from the NAT10 knockdown group, as well as in those from the groups treated with remodelin alone or remodelin combined with cisplatin (Fig. 2I).

To further validate the clinical relevance of our findings, we utilized the GEPIA3 database (https://gepia3.bioinfoliu.com) to confirm that high NAT10 expression tended to be associated with shorter progression-free survival (PFS) in gastric cancer patients receiving cisplatin-based chemotherapy (Sup. Fig. 4A). In the large cohort of unselected gastric cancer patients retrieved from the Kaplan-Meier Plotter database (https://www.kmplot.com/analysis/), we found a highly significant correlation between elevated NAT10 expression and poorer progression-free survival (PFS) (Sup. Fig. 4B). These results suggest that NAT10 may represent a promising therapeutic target for treating cisplatin-resistant tumors.

### NAT10 promoted the mRNA stability of DUSP1 and enhanced its expression in gastric cancer cells

**Fig. 3:**
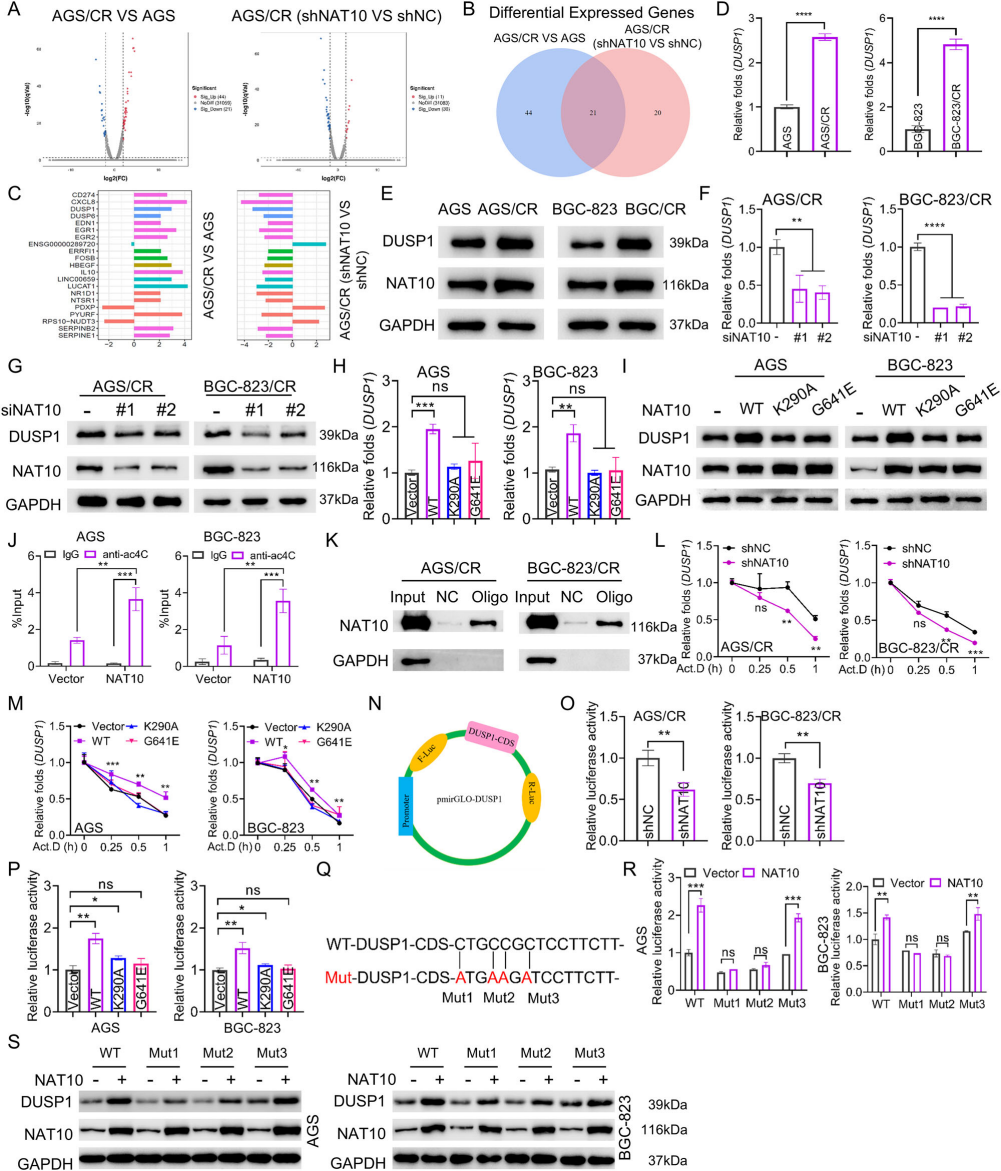
NAT10 promotes the mRNA stability of DUSP1 and enhances its expression in gastric cancer cells. **A** A volcano plot based on the transcriptomic sequencing results was used to compare the differentially expressed genes between AGS cells and AGS/CR control cells, as well as between AGS/CR control cells and AGS/CR cells with NAT10 knockdown. The inclusion criterion was log_2_FC (fold change)≥2. **B** A Venn diagram was used to illustrate the intersection of the differentially expressed genes between the AGS/CR group vs. the AGS group and the AGS/CR NAT10 shRNA group vs. the AGS/CR shNC group. **C** Bar chart of the log_2_ FC values of the 21 overlapping genes. **D**, **E** The expression level of DUSP1 was determined by RT-qPCR (**D**) and Western blot (**E**) in the parental cells and cisplatin-resistant cells. **F**, **G** The expression of DUSP1 was assessed by RT-qPCR (**F**) and Western blot (**G**) in cisplatin-resistance cells transfected with NAT10 siRNAs. **H**, **I** The expression level of DUSP1 was assessed by RT-qPCR (**H**) and Western blot (**I**) in AGS and BGC-823 cells transfected with empty vector, WT-NAT10 expression vector, the K290A or G641E NAT10 mutant. **J** acRIP-qPCR was used to detect the enrichment of DUSP1 mRNA using ac4C antibody in the gastric cancer cells with NAT10 overexpression. **K** RNA pull-down assay was used to detect the interaction between DUSP1mRNA and NAT10 by adding the DUSP1-CDS probe or control probe to AGS/CR or BGC-823/CR cells. The pulled-down protein was detected by Western blot. **L** The stability of DUSP1 mRNA was detected by RT - qPCR in AGS/CR and BGC - 823/CR cells transfected with shNAT10 or negative control, followed by treatment with Act. D for 0 h, 0.25 h, 0.5 h, and 1 h. **M** The stability of DUSP1 mRNA was detected by RT-qPCR in AGS and BGC-823 cells transfected with an empty vector, wild-type NAT10 expression vector, NAT10 K290A mutant vector, or NAT10 G641E mutant vector, followed by treatment with Act. D for 0 h, 0.25 h, 0.5 h, and 1 h. **N** A schematic illustration to show the construction of the dual luciferase reporter plasmid by inserting the DUSP1 CDS sequence between the firefly luciferase (F-luc) and Renilla luciferase (R-Luc). **O** The dual-luciferase reporter activity was measured in cisplatin-resistant cells co-transfected with the DUSP1 luciferase reporter gene vector, along with shNAT10 or negative control. **P** The dual-luciferase reporter activity was measured in AGS or BGC-823 cells co-transfected with DUSP1 luciferase reporter gene vector, along with either the wild-type NAT10 (WT-NAT10) or mutant NAT10 expression vector. **Q** A schematic illustration to show the mutation sites of mutant DUSP1 luciferase reporter vectors. **R** The dual-luciferase reporter activity was measured in AGS and BGC-823 cells that were co-transfected with the wild-type DUSP1 or mutant DUSP1 luciferase reporter vector, along with the empty vector or NAT10 expression vector. **S** Western blot analysis was performed to assess the expression of DUSP1 in AGS or BGC-823 cells transfected with wild-type or ac4C site-mutated DUSP1 expression vector (CDS-Mut1, CDS-Mut2, or CDS-Mut3), together with an empty vector or a NAT10 expression vector. *p < 0.05, **p < 0.01, *** *p* < 0.001, **** *p* < 0.0001 and ns, no significant.

To further explore the underlying mechanisms by which NAT10 promotes cisplatin resistance of gastric cancer cells, we carried out a transcriptomic sequencing to identify differentially expressed genes among AGS cells, AGS/CR control cells, and AGS/CR cells with NAT10 knockdown (Fig. 3A). Biological Process Gene Ontology (GO) analysis of the differentially expressed genes indicated a significant enrichment of the apoptosis pathway, suggesting that NAT10 may mediate cisplatin resistance by enhancing the resistance of gastric cancer cells to apoptosis (Sup. Fig. 5). We identified 65 differentially expressed genes in the comparison between the AGS/CR group and the AGS group, and 41 differentially expressed genes in the comparison between the AGS/CR-NAT10 shRNA group and the AGS/CR-NC group. By performing an intersection analysis of the differentially expressed genes from these two datasets, we identified 21 overlapping genes (Fig. 3B, C). Among these, 16 are protein-coding genes. we then assessed the expression levels of the 16 genes in AGS and BGC-823 cells using RT-qPCR and confirmed that seven genes (including CD274, CXCL8, DUSP1, FOSB, HBEGF, SERPINB2 and SERPINE1) are upregulated in cisplatin-resistant cells and downregulated by NAT10 shRNA (Sup. Fig. 6). Based on previous reports, among these seven genes, dual specificity phosphatase 1 (DUSP1) is known to mediate cisplatin resistance in cancer cells [34–36]. Additionally, a highly conserved acetylation sites on DUSP1 mRNA was predicted using the PACES tool (Sup. Fig. 7) [37]. Therefore, we focused on DUSP1 for further investigation. We found that DUSP1 mRNA and protein levels were significantly increased in cisplatin-resistant cells (Fig. 3D, E). We then transfected NAT10 siRNAs into AGS/CR and BGC-823/CR cells, or treated the cells with NAT10 inhibitor remodelin, and then determined the expression level of DUSP1. The results showed that NAT10 knockdown or remodelin treatment reduced DUSP1 levels (Fig. 3F, G and Sup. Fig. 8A, B). Additionally, we transfected gastric cancer cells with WT-NAT10 or mutant NAT10 expression vectors and assessed DUSP1 expression. The results demonstrated that overexpression of WT-NAT10, but not mutant NAT10, increased DUSP1 expression (Fig. 3H, I), indicating that the acetyltransferase activity of NAT10 is crucial for this regulation. We further performed an acRIP-qPCR assay to investigate whether NAT10 mediates acetylation modification of DUSP1. The results confirmed that NAT10 overexpression increased the enrichment of DUSP1 mRNA by the ac4C antibody in AGS and BGC-823 cells (Fig. 3J). To further explore whether NAT10 interacts with DUSP1 mRNA, we conducted an RNA pull-down assay. The results showed that the DUSP1 probe could enrich NAT10 in AGS/CR and BGC-823/CR cells, whereas the negative control probe could not (Fig. 3K), indicating a specific interaction between DUSP1 mRNA and NAT10. Subsequently, we investigated whether NAT10 affects the stability of DUSP1 mRNA by adding the transcription inhibitor actinomycin D (Act.D) to NAT10-knockdown or remodelin-treated gastric cancer cells at various time points. Our results demonstrated that NAT10 knockdown or remodelin treatment decreased the stability of DUSP1 mRNA (Fig. 3L and Sup. Fig.8C). In contrast, overexpression of WT-NAT10, but not mutant NAT10, enhanced the stability of DUSP1 mRNA (Fig. 3M). The potential ac4C site in DUSP1 mRNA predicted by the PACES tool with the highest score is located within the 327-341 region of the DUSP1 coding sequence (CDS). We therefore constructed a recombinant dual-luciferase reporter plasmid containing the DUSP1-CDS fragment (Fig. 3N) and transfected it into gastric cancer cells with NAT10 knockdown or overexpression. The results of dual-luciferase activity assay showed that NAT10 knockdown reduced the luciferase activity of the construct containing the DUSP1-CDS fragment (Fig. 3O). In contrast, overexpression of WT-NAT10, but not mutant NAT10, increased the luciferase activity (Fig. 3P). To further identify the specific sites of DUSP1 acetylated by NAT10, we constructed various mutant luciferase reporter vectors in which the predicted potential acetylation sites within DUSP1-CDS region were mutated (Fig. 3Q). The results of the dual luciferase activity assay showed that both dual mutations at positions 330 and 331, and a single mutation at position 327 in the DUSP1-CDS region, could reverse the regulation of WT-DUSP1 luciferase activity by NAT10 (Fig. 3R). In addition, we constructed three DUSP1 expression vectors harboring mutations at the identified positions. We then co-transfected gastric cancer cells with either WT-DUSP1 or different DUSP1 mutants, together with empty vector or NAT10 expression vector. Western blot results showed that Mutant 1 (site C327) and Mutant 2 (site C330 and C331) partially reversed the NAT10 overexpression-mediated upregulation of DUSP1, further confirming that positions C327, C330, and C331 within the DUSP1 CDS are potential modification sites (Fig. 3S).

### DUSP1 is required for NAT10-induced cisplatin resistance in gastric cancer

**Fig. 4:**
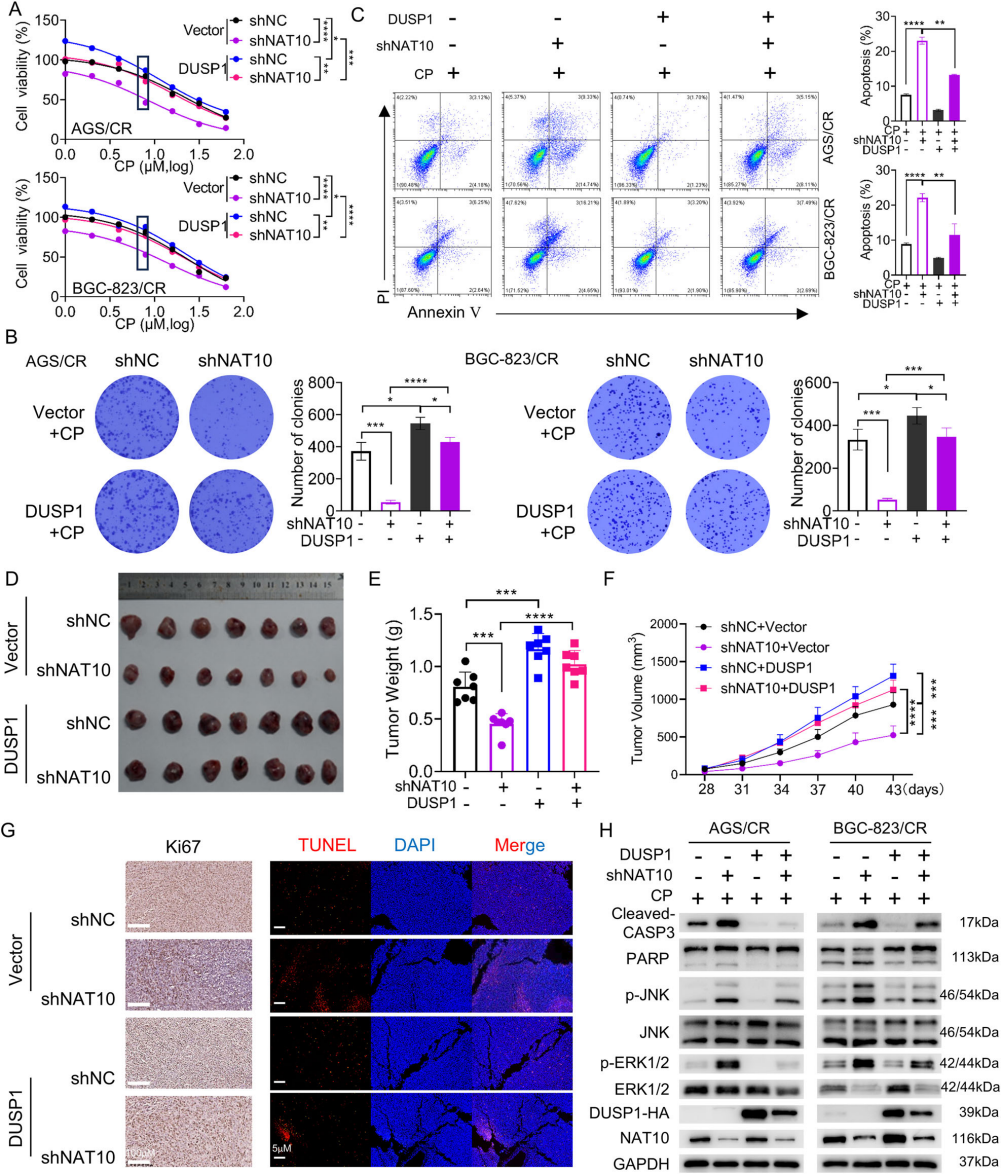
DUSP1 is required for NAT10-induced cisplatin resistance in gastric cancer. **A**–**C** Cell viability, clonogenic capacity and cell apoptosis were evaluated using CCK-8 assays (**A**) and colony formation assays (**B**) and flow cytometry (**C**) in AGS/CR and BGC-823/CR cells with indicated treatment. **D** The representative images of tumors from the indicated grafted mice treated with cisplatin. **E**, **F** Statistical analysis of the weight (**E**) and volume (**F**) of the tumors in (**D**). **G** Representative images of Ki67 expression detected by IHC and cell apoptosis determined by TUNEL assay in tumors. **H** Expression of cleaved- Caspase3, PARP and phosphorylated ERK and JNK were analyzed by Western blot in AGS/CR and BGC-823/CR with different transfection. *p < 0.05, **p < 0.01, *** *p* < 0.001, **** *p* < 0.0001 and ns, no significance.

Having established the regulatory effect of NAT10 on DUSP1, we next sought to explore whether DUSP1 is involved in NAT10-induced cisplatin resistance in gastric cancer. By evaluating the effects of cisplatin treatment on cell viability, colony formation ability, and apoptosis rate in AGS/CR and BGC-823/CR cells co-transfected with NAT10 siRNA and DUSP1 overexpression vector, we found that DUSP1 overexpression partially reversed the cisplatin chemosensitivity mediated by NAT10 knockdown (Fig. 4A–C). Additionally, DUSP1 overexpression could also reversed the cisplatin chemosensitivity mediated by remodelin (Sup. Fig. 9A, B).

To further verify the effect of DUSP1 on cisplatin chemosensitivity in vivo, we established a chemotherapy mouse model. Specifically, we subcutaneously injected mice with four groups of cells: BGC-823/CR control cells, BGC-823/CR cells with NAT10 knockdown, BGC-823/CR cells with DUSP1 overexpression, and BGC-823/CR cells with both NAT10 knockdown and DUSP1 overexpression, followed by cisplatin treatment. As expected, compared to the control group, the tumor weight and volume in NAT10 knockdown group are significantly reduced. In contrast, DUSP1 overexpression increased tumor weight and volume and reversed the cisplatin sensitivity induced by NAT10 knockdown (Fig. 4D–F). In addition, compared with the control group, the NAT10 knockdown group showed a significant decrease in Ki67-positive cells and an increase in TUNEL positive cells. In contrast, DUSP1 overexpression not only significantly increased the number of Ki67 positive cells and decreased the number of TUNEL positive cells, but also reversed the effects mediated by NAT10 knockdown (Fig. 4G). Previous studies have shown that DUSP1 decreases JNK and ERK phosphorylation and exerts an anti-apoptotic role via the JNK and ERK signaling pathways [38–40]. Here, we confirmed that DUSP1 overexpression reduced the cleavage of PARP and caspase-3, as well as the phosphorylation levels of JNK and ERK. Additionally, it reversed the NAT10 knockdown-mediated increase in PARP and caspase-3 cleavage and JNK and ERK phosphorylation (Fig. 4H). These findings demonstrate that DUSP1 is involved in NAT10-mediated cisplatin resistance via JNK and ERK signaling pathways in gastric cancer cells.

### NAT10 enhances PD-L1 expression in gastric cancer

**Fig. 5:**
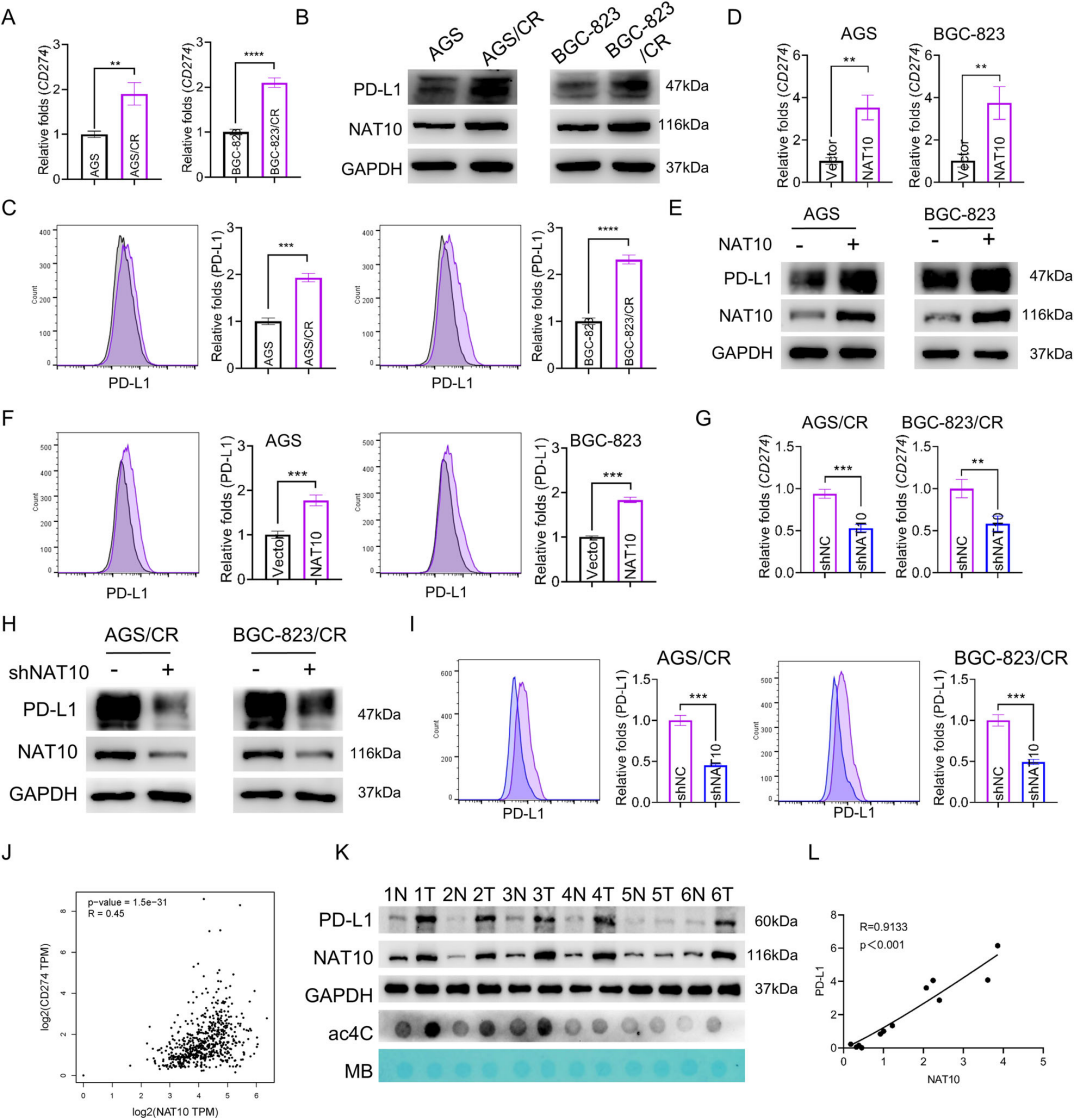
NAT10 enhances PD-L1 expression in gastric cancer. **A**, **B** RT- qPCR (**A**) and Western blot (**B**) were used to detect the expression of PD-L1 in parental cells and cisplatin-resistant cells. **C** Flow cytometry was used to detect PD-L1 level on the surface of the parental cells and cisplatin - resistant cells. **D**, **E** RT- qPCR (**D**) and Western blot (**E**) were used to detect the expression of PD-L1 in AGS and BGC-823 cells transfected with empty vector or NAT10 expression vector. **F** Flow cytometry was used to detect PD-L1 on the surface of the gastric cancer cells transfected with empty vector or NAT10 expression vector. **G**, **H** RT- qPCR (**G**) and Western blot (**H**) were used to detect the expression of PD-L1 in cisplatin-resistant gastric cancer cells transfected with negative control shRNA (shNC) or NAT10 shRNA (shNAT10). **I** Flow cytometry was used to detect PD-L1 on the surface of the cisplatin-resistant gastric cancer cells transfected with negative control shRNA (shNC) or NAT10 shRNA (shNAT10). **J** A correlation analysis of NAT10 and CD274 expression level on gastric cancer patients from the TCGA dataset. **K** Representative Western blot results of NAT10 and PD-L1 expression in gastric cancer tissues and adjacent non-tumor tissues. **L** Correlation analysis between NAT10 and PD-L1 protein abundance in gastric cancer tissues. Statistical significance was determined by a two-tailed unpaired Student’s t-test. One-way ANOVA was used to compare the means of three or more experimental groups. Error bars show the SDs from three independent experiments. *p < 0.05, **p < 0.01, *** *p* < 0.001, **** *p* < 0.0001 and ns, no significance.

The immune checkpoint molecule PD-L1 is currently recognized as one of the most critical targets in immunotherapy. High PD-L1 expression is frequently observed in drug-resistant tumor cells, which contributes to resistance to immunotherapeutic interventions [41–43]. Our transcriptomic sequencing data and qRT-PCR validation confirmed that the PD-L1-encoding gene CD274 is upregulated in cisplatin-resistant gastric cancer cells and downregulated upon NAT10 knockdown (Figs. 3C, 5A and Sup. Fig. 6). We further used western blot to detect the protein abundance of PD-L1 and flow cytometry to assess PD-L1 levels on the cell membrane. Our results indicated that, compared with parental cells, PD-L1 abundance was elevated in cisplatin-resistant gastric cancer cells (Fig. 5B), and the amount of PD-L1 protein localized to the cell membrane was also increased (Fig. 5C). Overexpression of NAT10 significantly increased CD274 mRNA levels, PD-L1 protein abundance, and membrane-localized PD-L1 protein levels (Fig. 5D–F). Conversely, NAT10 knockdown or treatment with the NAT10 inhibitor remodelin in cisplatin-resistant gastric cells reduced CD274 mRNA levels, PD-L1 protein abundance, and membrane-localized PD-L1 levels (Fig. 5G–I and Sup. Fig. 10A–C). In addition, we further explored the clinical relevance between NAT10 and PD-L1 expression levels. We found that the transcript of NAT10 is positively associated with CD274 in TCGA dataset (Fig. 5J). We further used western blot to detect the protein levels of NAT10 and PD-L1 in clinical gastric cancer samples and their corresponding adjacent non-tumor tissues, and found that, NAT10 and PD-L1 were highly expressed in most gastric cancer tissues compared with the matched non-tumor tissues (Fig. 5K). Regression analysis results showed a positive correlation between NAT10 and PD-L1 protein abundances in gastric cancer tissues (Fig. 5L).

### NAT10 regulates the expression level of PD-L1 at the transcriptional level through transcription factor FOSB

**Fig. 6:**
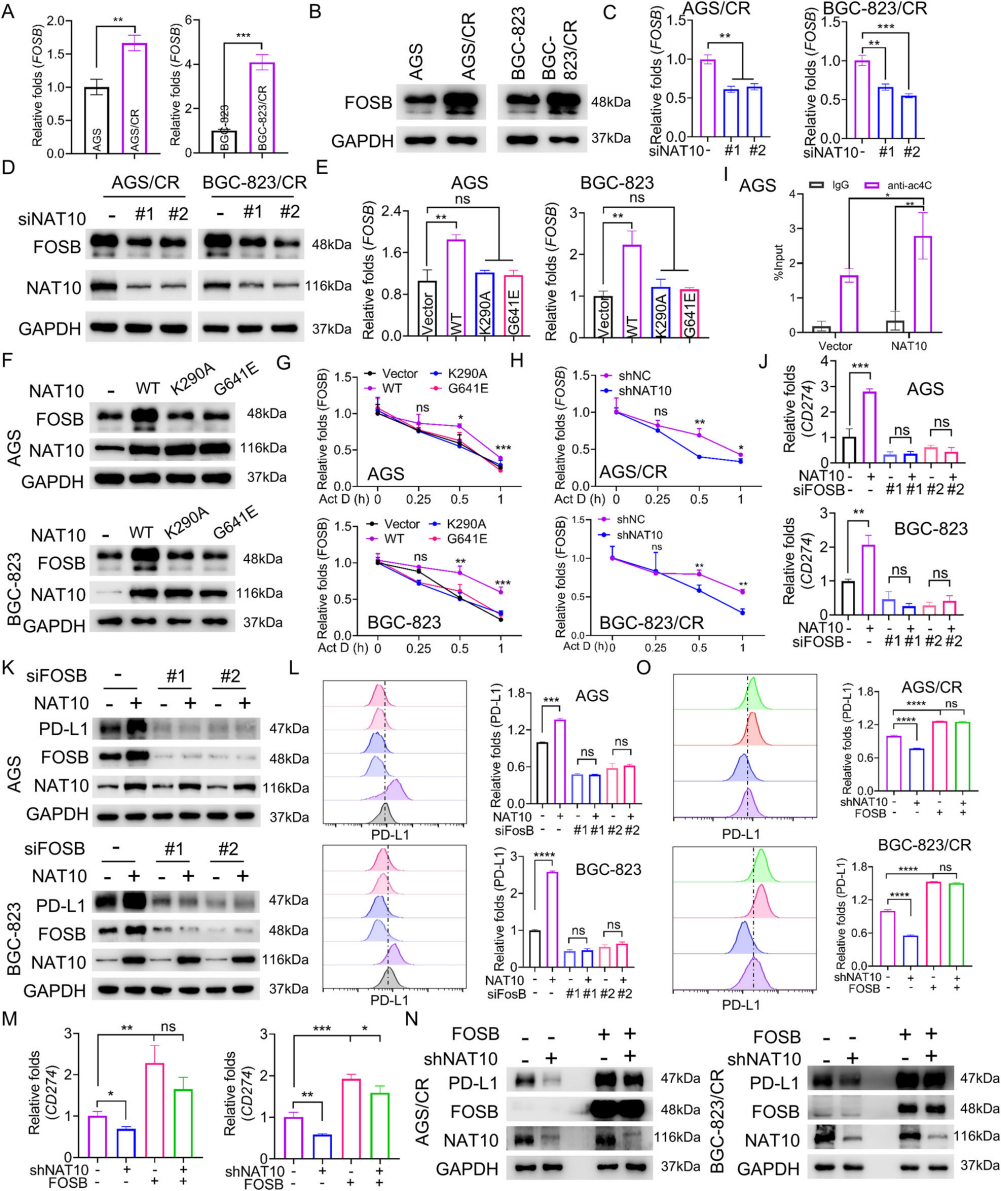
NAT10 regulates the expression level of PD-L1 at the transcriptional level through FOSB. **A**, **B** The expression of FOSB was detected using RT- qPCR (**A**) and Western blot (**B**) in parental cells and cisplatin resistant cells. **C**, **D** The expression level of FOSB was detected by RT-qPCR (**C**) and Western blot (**D**) in cisplatin-resistant gastric cancer cells which were transfected with control siRNA or NAT10 siRNA. **E**, **F** After transfecting AGS and BGC-823 cells with empty vector, wild-type NAT10 expression vector, or mutant NAT10 expression vector (K290A or G641E) for 48 h, the expression level of FOSB was detected by RT-qPCR (**E**) and Western blot (**F**). **G** The stability of FOSB mRNA was detected by RT - qPCR in AGS and BGC - 823 cells transfected with an empty vector, a wild - type NAT10 expression vector, NAT10 K290A mutant vector or NAT10 G641E mutant vector and then treated with Act D for 0 h, 0.25 h, 0.5 h, and 1 h. **H** The stability of FOSB mRNA was detected by RT - qPCR in AGS/CR and BGC - 823/CR cells transfected with NAT10 shRNA or netative control shRNA and then treated with Act D for 0 h, 0.25 h, 0.5 h, and 1 h. **I** acRIP-qPCR was used to detect the enrichment of FOSB mRNA using ac4C antibody in the gastric cancer cells with NAT10 overexpression. **J**, **K** The expression of PD-L1 was analyzed by RT-qPCR (**J**) and Western blot (**K**) in AGS and BGC-823 cells with different co-transfection. **L** Flow cytometry was used to detect the level of PD-L1 on the surface of AGS and BGC-823 cells with different co-transfection. **M**, **N** The expression of PD-L1 was analyzed by RT-qPCR (**M**) and Western blot (**N**) in AGS/CR and BGC-823/CR cells with different co-transfection. **O** Flow cytometry was used to detect the level of PD-L1 on the surface of AGS/CR and BGC-823/CR cells with different co-transfection. Statistical significance was determined by a two-tailed unpaired Student’s t-test. One-way ANOVA was used to compare the means of three or more experimental groups. Error bars show the SDs from three independent experiments. *p < 0.05,**p < 0.01, *** *p* < 0.001, **** *p* < 0.0001 and ns, no significant.

To further elucidate the mechanism by which NAT10 regulates PD-L1, we first used the PACES tool to predict the presence of potential ac4C sites in CD274 mRNA, but we did not find any potential ac4C sites [37]. Consistent with this, acRIP-qPCR results revealed no enrichment of CD274 mRNA by the ac4C antibody. Therefore, we speculated that NAT10 may regulate the expression of PD-L1 through modulating its upstream transcription factors. Based on our RNA-seq results, we noticed that the expression level of transcription factor FOSB was increased in AGS/CR cells, and NAT10 knockdown reduced its level (Fig. 3C). So, we speculate that FOSB may be a potential downstream target gene of NAT10 to regulate the transcriptional of PD-L1. We used RT-qPCR and western blot to confirm that FOSB was significantly upregulated in cisplatin-resistant cells compared with parental cells (Fig. 6A, B). Additionally, NAT10 knockdown reduced FOSB levels in cisplatin-resistant cells (Fig. 6C, D), and the NAT10 inhibitor remodelin treatment exerted a similar effect (Sup.Fig.11A-B). Conversely, overexpression of WT-NAT10, but not its enzymatically inactive mutant, upregulated FOSB expression (Fig. 6E, F). Moreover, overexpression of WT-NAT10, rather than the mutant form, increased FOSB stability (Fig. 6G), whereas NAT10 knockdown or remodelin treatment promoted FOSB degradation (Fig. 6H and Sup. Fig. 11C). We further performed an acRIP-qPCR assay to investigate whether NAT10 mediates acetylation modification of FOSB. The results confirmed that NAT10 overexpression increased the enrichment of FOSB mRNA by the ac4C antibody in AGS cells (Fig. 6I).

To clarify whether NAT10 regulates the expression of PD-L1 via FOSB, we conducted rescue experiments. As shown in Fig. 6J–L, FOSB knockdown reduced PD-L1 expression and reversed the NAT10 overexpression-mediated upregulation of PD-L1. Conversely, FOSB overexpression increased PD-L1 expression and abolished the NAT10 knockdown-mediated downregulation of PD-L1 (Fig. 6M–O). These results suggest that NAT10 regulates PD-L1 expression via transcription factor FOSB.

To further investigate whether FOSB regulates PD-L1 expression at the transcriptional level, we performed a dual-luciferase reporter assay. The results showed that FOSB knockdown decreased the luciferase reporter activity of the PD-L1 (CD274) gene promoter, while FOSB overexpression increased the luciferase activity of the CD274 promoter (Sup. Fig. 12A and B). Based on predictions from JASPAR software, a potential FOSB binding site (-288 ~ -297) was identified within the CD274 promoter. We then mutated this potential binding site in the CD274 promoter, and subsequent dual-luciferase assays revealed that FOSB overexpression no longer affected the activity of the mutated luciferase reporter (Sup. Fig. 12B). Additionally, ChIP-qPCR confirmed that the HA antibody enriched the CD274 promoter region in AGS and BGC-823 cells transfected with the HA-tagged FOSB expression vector (Sup. Fig. 12C). These results demonstrate that FOSB binds to the CD274 promoter and regulates its expression.

### Inhibition of NAT10 increases the sensitivity of immunotherapy

**Fig. 7:**
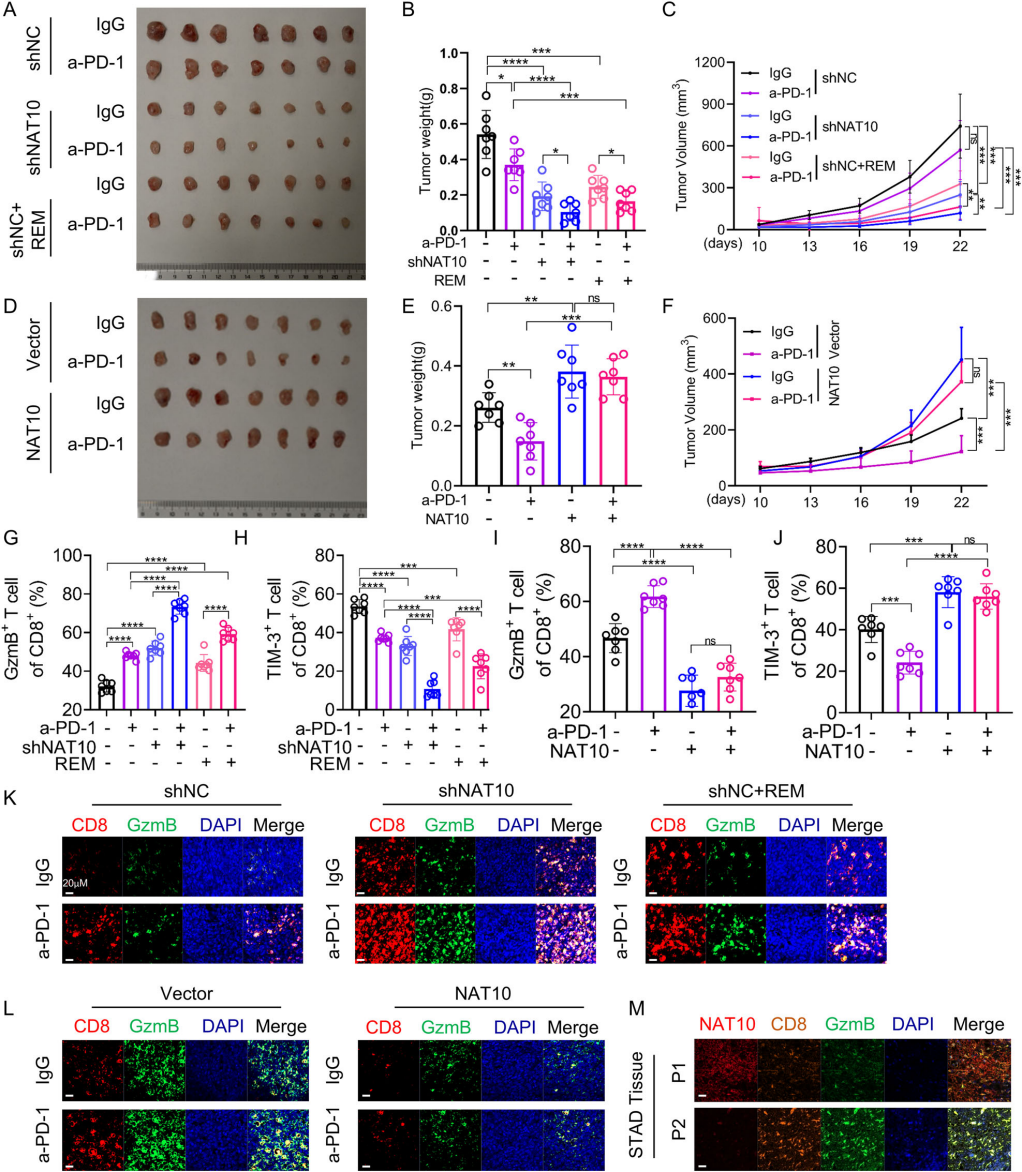
Inhibition of NAT10 increases the sensitivity of immunotherapy. **A** The subcutaneous tumors in C57BL/6 mice are presented. These mice were injected with MFC/CR cells containing shNC or sh-NAT10 and then treated with a PD-1 antibody alone or PD-1 antibody plus remodelin. **B**, **C** Statistical analysis of the tumor weight (**B**) and the tumor volumes (**C**) (*n* = 7). **D** The subcutaneous tumors of C57BL/6 mice injected with control MFC/CR cells or MFC/CR cells carrying NAT10 expression vector and then treated with PD-1 antibody are shown. **E**, **F** Statistical analysis of the tumor weight (**E**) and the tumor volumes (**F**) (*n* = 7). **G–J** The percentages of tumor - infiltrating CD8⁺ GzmB⁺ T cells (**G**, **I**) and CD8⁺Tim3⁺ T cells (**H**, **J**) in the tumors from (**A**, **D**) were analyzed via flow cytometry. **K**, **L** The expression of CD8^+^ and GzmB^+^ T cells infiltrating the tumor was detected by immunofluorescence in the tumors from (**A**, **D**). **M** The expression of NAT10, CD8^+^ and GzmB^+^ T cells infiltrating the tumor was detected by immunofluorescence in human gastric cancer tissues. Statistical significance was determined by a two-tailed unpaired Student’s t-test. One-way ANOVA was used to compare the means of three or more experimental groups. Error bars show the SDs from three independent experiments. *p < 0.05,**p < 0.01, *** *p* < 0.001, **** *p* < 0.0001 and ns, no significant.

Having clarified the regulatory role of NAT10 in PD-L1 expression, we sought to determine whether NAT10 inhibition could abrogate PD-L1-mediated immune escape in gastric cancer and enhance the sensitivity to immunotherapy. We established a cisplatin-resistant cell line (MFC/CR) from the mouse gastric cancer cell MFC by continuously increasing cisplatin concentration. As shown in Sup.Fig.13A, the IC_50_ value of the MFC/CR cell line was significantly increased. We further confirmed that both NAT10 and PD-L1 were highly expressed in the cisplatin-resistant MFC/CR cells (Sup.Fig. 13B), which is consistent with the findings in human gastric cancer cell lines. To investigate whether NAT10 knockdown can enhance the immunosuppressive effect of PD-1 antibody therapy, we injected control MFC/CR cells (MFC-shNC) or NAT10-knockdown MFC/CR cells (MFC/CR-shNAT10) into immunocompetent C57BL/6 J mice. Subsequently, mice injected with control MFC/CR cells were treated with IgG antibody alone, PD-1 antibody alone, or a combination of the NAT10 inhibitor remodelin with either IgG antibody or PD-1 antibody. Mice injected with MFC/CR-shNAT10 cells were treated with IgG antibody alone or PD-1 antibody alone. The results showed that compared with IgG treatment group, PD-1 antibody treatment slightly reduced the tumor weight and volume in mice injected with MFC/CR-shNC cells, whereas it sharply reduced the tumor weight and volume in mice injected with MFC/CR-shNAT10 cells. Furthermore, tumor weight and volume were significantly lower in mice treated with the combination of PD-1 antibody and remodelin than in those treated with PD-1 antibody alone (Fig. 7A–C). Next, we injected MFC cells harboring empty vector (MFC-vector) or NAT10 expression vector (MFC-NAT10) into C57BL/6 J mice, which were then treated with either IgG antibody or PD-1 antibody. The results showed that compared with the IgG treatment group, PD-1 antibody treatment significantly reduced the tumor weight and volume in mice injected with MFC-vector cells. However, PD-1 antibody treatment had almost no effect on the tumor weight and volume in mice injected with MFC cells with NAT10 overexpression (Fig. 7D–F). Subsequently, we used the tumor tissues from the above experiments to further detect the proportion of CD8^+^ T cells infiltration into the tumor and investigate the activity of CD8^+^ T cells through determining the expression of the activation marker granzymeB (GzmB) and the exhaustion marker Tim3^+^ in CD8^+^ T cells by flow cytometry. The results showed that NAT10 knockdown or remodelin treatment increased the proportion of CD8^+^ T cells infiltration into the tumor and enhanced their activity, with an increased expression of the activation marker GzmB and a decreased expression of the exhaustion marker Tim3 (Fig. 7G, H and Sup. Fig. 14). On the contrary, overexpression of NAT10 decreased the proportion of CD8^+^ T cells infiltrating the tumor and weakened their activity, with a reduced expression of the activation marker granzyme B(GzmB) and an increased expression of the exhaustion marker Tim3 (Fig. 7I, J and Sup. Fig. 14). Multiplex immunofluorescence staining further showed that NAT10 knockdown or remodelin treatment increased the proportion of CD8^+^ T cells infiltration into the tumor and the expression of GzmB (Fig. 7K), whereas overexpression of NAT10 reduced the proportion of CD8^+^ T cells infiltration into the tumor and the expression of GzmB (Fig. 7L). In addition, we used clinical gastric cancer samples to detect the expression of NAT10, CD8 and GzmB through multiplex immunofluorescence staining. The results showed that the high expression of NAT10 in the patient tissues was accompanied by the low expression of CD8 and GzmB, which is consistent with the results in the mice (Fig. 7M).

## Discussion

Gastric cancer is one of the common malignant tumors in the world [1]. Systemic chemotherapy is the most common treatment method for patients with advanced gastric cancer. Cisplatin, a standard chemotherapeutic drug for gastric cancer, shows effective anti-tumor effects. However, patients often develop acquired resistance during cisplatin treatment. They are not only insensitive to cisplatin treatment, but also exhibit immune escape, thus becoming resistant to immunotherapy. Finding effective ways to overcome cisplatin resistance is a crucial step in the treatment of gastric cancer. Epigenetic modification is one of the new directions for exploring drug resistance mechanisms at present. Among them, N4-acetylcytidine (ac4C), a significant post-transcriptional RNA modification in eukaryotes, is essential for mRNA stability and translational control. NAT10 is the only enzyme that catalyzes the modification of ac4C mRNA, playing an important role in tumorigenesis, metastasis and invasion, as well as in chemotherapy resistance [28–30]. Existing evidence shows that NAT10 exerts conserved roles in driving chemoresistance and immune escape via context-dependent molecular mechanisms across a spectrum of solid tumors, with its ac4C RNA modification activity serving as a core functional axis in most cases. Specifically, in triple-negative breast cancer, the NAT10/ac4C/JunB signaling cascade fosters malignant progression and immunosuppression by reprogramming tumor cell metabolism toward glycolysis addiction [31], Additionally, NAT10-dependent inhibition of RAD51 N4-acetylcytidine modification enhances olaparib sensitivity in breast cancer [44]. In nasopharyngeal carcinoma, NAT10’s acetyltransferase activity suppresses anti-tumor T-cell immunity and accelerates tumor progression through the DDX5/HMGB1 axis [45], and pharmacological inhibition of NAT10 reverses sorafenib resistance by triggering ac4C-dependent ferroptosis [46]. In pancreatic ductal adenocarcinoma, NAT10 drives tumor progression and immune evasion via the N4-acetylated LAMB3-mediated FAK/ERK pathway [47], and also contributes to intrinsic resistance to gemcitabine, a first-line chemotherapeutic for PDAC [48]. However, the role and mechanism of NAT10 in cisplatin resistance and immune escape in gastric cancer remain unclear.

In this study, we demonstrated for the first time that the expression level of NAT10 and the total ac4C modification level are significantly higher in cisplatin-resistant gastric cancer cells compared to their cisplatin-sensitive counterparts. Knockdown of NAT10 enhanced cisplatin sensitivity in cisplatin-resistant gastric cancer cells, whereas overexpression of NAT10 decreased cisplatin sensitivity in cisplatin-sensitive gastric cancer cells. We further confirmed that NAT10 can regulate the expression level of the dual-specificity phosphatase DUSP1 by maintaining the stability of DUSP1 mRNA. DUSP1 belongs to the serine/threonine dual specificity phosphatase family members. It can regulate multiple signaling pathways, such as ERKs, p38MAPK, and JNK, and is involved in normal cell growth, differentiation, and apoptosis, as well as the development of chemoresistance [34]. We confirmed that DUSP1 decreased the sensitivity of cisplatin-resistant gastric cancer cells to cisplatin, reduced the phosphorylation of ERK and JNK, and diminished the cleavage of caspase-3 and PARP, and is involved in NAT10-mediated cisplatin resistance. We further used acRIP-qPCR and RNA-pulldown assay to validate that NAT10 can interact with DUSP1 mRNA and mediate the ac4C modification of DUSP1. The acetylation site of DUSP1 was identified within its coding sequence (CDS) region via luciferase reporter assay and site-directed mutagenesis. Moreover, the acetyltransferase activity of NAT10 are crucial for mediating ac4C modification of DUSP1 mRNA and maintaining the stability of DUSP1 mRNA.

Studies have reported that when chemotherapy resistance emerges in patients during treatment, the damage to the immune system caused by chemotherapeutic drugs leads to increased expressions of programmed death-ligand 1 (PD-L1) and cytotoxic T-lymphocyte-associated antigen 4 (CTLA4), which in turn results in resistance to immunotherapy in the advanced stage [49, 50]. In this study, we found that NAT10 overexpression leads to increased PD-L1 expression, while NAT10 knockdown reduces PD-L1 levels. Knocking-down NAT10 or using a NAT10 inhibitor can not only enhance the chemosensitivity of cisplatin-resistant gastric cancer cells to cisplatin via the JNK and ERK pathways mediated by DUSP1, but also increase the sensitivity of these cells to PD-1 immunotherapy and promote the infiltration of tumor-infiltrating immune cells. Additionally, we evaluated the clinical relevance of NAT10-mediated PD-L1 regulation and found that NAT10 expression is positively correlated with PD-L1 expression in clinical gastric cancer tissue specimens. Therefore, the use of a NAT10 inhibitor may be a promising option for patients with advanced gastric cancer, as it can not only enhance the efficacy of chemotherapy but also increase the sensitivity of drug-resistant gastric cancer cells to PD-1 immunotherapy.

The regulation of PD-L1 mRNA level is a complex process involving multiple levels and mechanisms. Many transcription factors can bind to the promoter region of the PD-L1 gene to regulate its transcription [51, 52]. For example, NF-κB (nuclear factor κB) can recognize and bind to specific sequences on the promoter of the PD-L1 gene, promoting the transcription of the PD-L1 gene into mRNA and playing an important role in inflammatory responses and tumor immune escape in various cell types [53]. Similarly, AP-1 (activator protein 1) can also bind to the PD-L1 promoter region to positively regulate the transcription of PD- L1 [54, 55]. In addition, the mRNA level of PD - L1 can also be regulated at the post-transcriptional level. For example, METTL3 can recognize the specific sequences on PD-L1 mRNA and mediate the N6-methyladenosine (m6A) modification of PD-L1 mRNA [56]. This modification can recruit specific RNA-binding proteins that protect the mRNA from degradation by nucleases, thereby increasing the stability of PD-L1 mRNA in various tumor types. In this study, we investigated whether NAT10 can mediate the ac4C modification of PD-L1 at the post-transcriptional level using database prediction and acRIP-qPCR assays. However, we did not find evidence that NAT10 mediates the ac4C modification of PD-L1 mRNA. Instead, we confirmed that NAT10 regulates PD-L1 expression via FOSB. NAT10 mediates the ac4C modification of FOSB, enhances its stability, and FOSB binds to the promoter of the PD-L1 gene (CD274) to increase its expression at the transcriptional level. It is well established that FOSB can form the AP-1 complex with members of the JUN protein family, which in turn enhances the transcriptional activity of gene promoters containing the AP-1 consensus sequence 5’-TGA [GC] TCA-3’ [57, 58]. Whether FOSB requires complex formation with JUN proteins to cooperatively bind to the PD-L1 promoter and regulate its expression in gastric cancer cells requires further investigation.

**Fig. 8:**
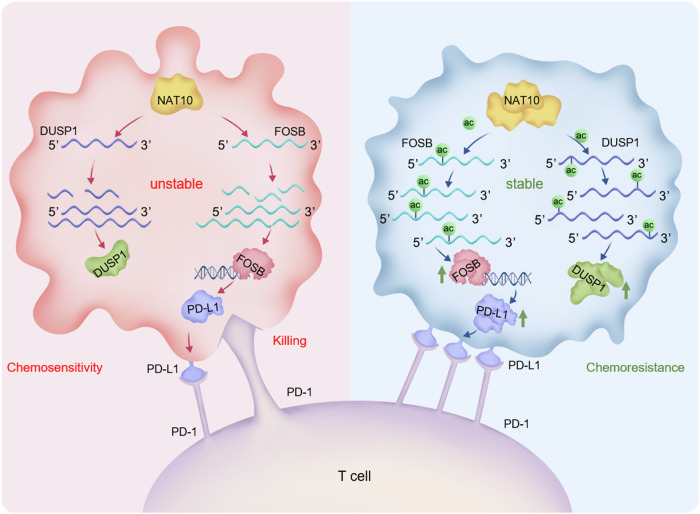
Proposed model of NAT10 promotes cisplatin resistance and immune escape by increasing the expression of DUSP1 and PD-L1 in gastric cancer.

In summary, we confirmed that NAT10 increases the protein level of DUSP1 by inducing the ac4C modification of DUSP1 mRNA and enhancing the stability of DUSP1 mRNA, thereby enhancing the resistance of gastric cancer cells to cisplatin. Additionally, NAT10 can indirectly increase the expression of PD-L1 through FOSB, promoting the immune escape of cisplatin resistant gastric cancer cells (Fig. 8). Therefore, targeting NAT10 may serve as an effective therapeutic strategy for gastric cancer by overcoming cisplatin resistance and immunosuppression.

## Materials and methods

### Cell culture and the establishment of cisplatin-resistant gastric cancer cells

Human gastric cancer cell lines AGS, BGC-823 were stored in our laboratory. Mouse forestomach carcinoma cell line (MFC) was purchased from Procell Life Science& Technology Co., Ltd. (Wuhan, China). Cisplatin-resistant gastric cancer cells (AGS/ CR, BGC-823/CR and MFC/CR) were established via an induction method of gradually increasing cisplatin (Cat# HY-17394, MCE) concentrations. AGS and AGS/CR were routinely cultured in F-12 medium (Cat# CM10080, Macgene) supplemented with 10% fetal bovine serum (Cat# 164210-50, Pricella). BGC-823 and MFC were cultured in RPIM-1640 medium (Cat# 01-100-1 A, Biological Industries) supplemented with 10% fetal bovine serum. Cisplatin was added into the cisplatin- resistant cells to maintain the drug resistance. All of the cells were supplemented with 1% penicillin–streptomycin (Cat# C100C5, NCM Biotech) and cultured at 37 °C in a humidified incubator with 5% CO_2_.

### Clinical samples

Human gastric cancer specimens and matched adjacent non-tumorous tissues were acquired from the Cancer Hospital of Shandong. The clinicopathological information of the samples is listed in Suppl. Table 1. All the experiments were approved by the ethics committee of the School of Basic Medical Sciences, Shandong University (ECSBMSSDU2023-1-25).

### siRNAs and plasmids

The small interfering RNAs (siRNAs) specifically targeting NAT10, DUSP1, and FOSB, as well as the negative control siRNA, were purchased from Genepharma (Shanghai, China). The sequences of the siRNAs are listed in Suppl. Table 2. The overexpression plasmids of NAT10, DUSP1, FOSB and CD274 promoter were purchased from Miao Ling Plasmid Platform (Wuhan, China). The NAT10 mutant plasmids were constructed using a PCR-based approach with designed primers (Suppl. Table 3) and the ClonExpress Ultra One Step Cloning Kit (Vazyme, Cat# C115), following the manufacturer’s protocol.

### Cell counting kit-8 (CCK-8) assay

Cells were plated in 96-well plates (5 × 10^3^ per well) and allowed to adhere for 24 h prior to treatment with cisplatin (Cat#HY-17394; MedChemExpress) at the indicated concentrations. At the end of the treatment, 10 µL of CCK-8 reagent (Cat#K1018; ApexBio) was added directly to each well containing 100 µL of medium. Cell viability was then assessed by measuring the absorbance at 450 nm with a reference wavelength of 650 nm.

### RNA isolation and RT-qPCR

Total RNA was extracted from gastric cancer cells using TRIzol reagent (Cat# R401 -01-AA, Vazyme,) according to the manufacturer’s instructions. Subsequently, the RNA was used as a template for reverse transcription (RT) with the PrimeScript RT kit (Cat# AG12204, Agilent Technologies). qPCR was conducted using a standard ChamQ Universal SYBR qPCR Master Mix (Cat# AG11701, Agilent Technologies). The relative expression of target genes was calculated using the 2^^(-ΔΔCt)^ method, with the expression of β-actin serving as the internal control. All specific primers are listed in Suppl. Table 4.

### Western blot

Total proteins were extracted from the cells by adding a lysis buffer (25 mM Tris, pH 8.0, 150 mM NaCl, 1% SDS, 1 mM EDTA,5%glycerol) containing protease inhibitor cocktail (Cat# HY-K0010, MCE), followed by sonication and centrifugation. Protein samples were separated by SDS-PAGE and transferred to PVDF membranes. Membranes were blocked with 5% non-fat milk for 1 h at room temperature, then incubated with primary antibodies overnight at 4 °C. After washing, membranes were incubated with HRP-conjugated secondary antibodies for 1 hour at room temperature. Protein bands were visualized using the Tanon Imaging Systerm(Shanghai Tanon Life Science Co.,Ltd). All antibodies used in this study are listed in Suppl.Table 5.

### RNA dot blot

Total RNA was extracted from cells using TRIzol reagent according to the manufacturer’s instructions and then diluted with RNase-free water. Subsequently, the diluted RNA was incubated at 95 °C for 3 min to disrupt its secondary structure. Then, 2 μL of the RNA was dropped onto a positively charged nylon membrane. The RNA was crosslinked to the membrane using ultraviolet light. After blocking with 5% milk, the membrane was incubated with the ac4C antibody (Cat# ab252215, Abcam) overnight at 4 °C. Next, the membrane was incubated with the secondary antibody, and visualized using the Tanon Imaging System (Shanghai Tanon Life Science Co., Ltd.). The membrane was stained with a methylene blue solution.

### Cell apoptosis analysis

The apoptosis rate of the gastric cells treated with cisplatin was detected by using the Annexin V-FITC/PI Apoptosis Kit (Cat# E-CK-A211,Elabscience). After cisplatin treatment, cells were harvested, washed with PBS, and counted. A total of 1 ~ 5 × 10⁵ cells were then re-pelleted,and resuspended in 500 μL of 1× Annexin V Binding Buffer. Subsequently, 5 μL of Annexin V-FITC and 5 μL of Propidium Iodide (PI) reagent (50 μg/mL) were added to the cell suspension. After vortexing, the mixture was incubated at room temperature for 20 minutes, protected from light. Samples were immediately analyzed by flow cytometry under light protection.

### mRNA stability assay

For mRNA stability assays, cells in 12-well plates were treated with 2.5 μg/mL actinomycin D (Act D) (Cat#HY-17559; MedChemExpress). Following incubation for designated durations (0, 0.25, 0.5, 1 h), total RNA was isolated using TRIzol reagent. Transcript abundance was determined by RT-qPCR (SYBR Green system) with β-actin as internal control.

### Colony formation assay

Treated gastric cancer cells (8×10^2^) were seeded into each well of a 12-well plate and then cultured in an incubator for 10 days. Cisplatin with a concentration of 20 μM was added once every three days. At the experimental endpoint, cells were fixed in absolute methanol and stained using 0.5% crystal violet ammonium oxalate solution (Cat #G1063; Solarbio).

### Dual-luciferase reporter assay

The cDNA fragment of the DUSP1-CDS was inserted between the luciferase and Renilla luciferase genes in the pmirGLO vector. The constructed pmirGLO-DUSP1-CDS vector was co-transfected into gastric cancer cells with the NAT10 expression vector or short hairpin RNA targeting NAT10 (shNAT10). After 48 h, the activities of firefly luciferase and Renilla luciferase were detected using the Dual-Luciferase® Reporter Assay System (Cat# E1960, Promega) according to the manufacturer’s instructions.

### RNA pull-down assay

Biotinylated DUSP1-CDS RNA was generated by using a 10 × Biotin RNA labeling mix (Cat# 1165597910, Roche) and a Transcript Aid T7 High Yield Transcription Kit (Cat# K0441, Thermo Fisher Scientific) according to the manufacturer’s instructions. The sequences of the T7-DUSP1-CDS are listed in Suppl. Table 3. RNA pull-down assays were performed by using an RNA pulldown kit (Cat# Bes5102, BersinBio) according to the manufacturer’s instructions. A total of 1 × 10⁷ cells were collected and lysed in 1.7 mL of lysis buffer. The supernatant was harvested, and a 100 μL aliquot was reserved as input. The remaining supernatant was split into two equal portions and incubated overnight at 4 °C with either negative control (NC) probes or DUSP1-Sense probes. The pulled-down proteins were subsequently analyzed by western blot.

### RNA sequencing (RNA-seq)

Extract RNAs from AGS cells, AGS/CR control cells, and AGS/CR cells with NAT10 knockdown. RNA-seq were performed by LC-BIOTECHNOLOGIES Co., Ltd. (Hang zhou, China).

### acRIP-qPCR

The total RNA (200 µg) was randomly digested into nucleotide chains of 100 to 200 bps using RNA Fragmentation Reagents (Cat# AM8740, Thermo Fisher Scientific). Then, a mixture of 5 µg ac4C antibody (Cat#ab252215, Abcam) and magnetic beads was incubated with the fragmented RNA. Subsequently, the RNAs were purified and analyzed by RT-qPCR.

### Detection of PD-L1 on the cell surface by flow cytometry

Gastric cancer cells that had been transfected with the NAT10 expression vector or NAT10 shRNA, or those that had been treated with remodelin, were digested with trypsin and then washed twice with PBS. Subsequently, these cells were incubated with a phycoerythrin conjugated anti-PD-L1 antibody (Cat#329706, BioLegend) for 30 min. Then, the stained cells are washed twice with pre-cooled PBS, and analyzed by flow cytometry. All the staining steps should be carried out in the dark.

### Lentiviral infection

The lentiviral-based shRNA used to knock-down the expression of human or mouse NAT10 was purchased from GenePharma (Shanghai,China). Then, we constructed NAT10 stable knockdown cells using shNAT10 for subsequent experiments.

### Animal experiments

Animal experiments in immunodeficient BALB/c nude mice and immunocompetent C57BL/6 mice (5-week-old, male) were performed. All the mice were purchased from GemPharmatech Co., Ltd (Jiangsu, China). For the experiment on nude mice, 5×10^6^ cisplatin-sensitive BGC-823 cells, cisplatin-resistant BGC-823/CR control cells and cisplatin-resistant BGC-823/CR cells with stable NAT10 knockdown were suspended in 100 ul of stroke-physiological saline solution (SPSS) and then subcutaneously injected into the right flanks of the mice to allow tumor formation. The nude mice injected with cisplatin-sensitive BGC-823 cells and cisplatin-resistant BGC-823/CR shRNA cells were divided into two groups, the SPSS group and the cisplatin (3 mg/kg, once every 4 days) treatment group. The mice injected with cisplatin-resistant BGC-823/CR control cells were divided into four groups, namely the SPSS group, the cisplatin treatment group (3 mg/kg, once every 4 days), the NAT10 inhibitor remodelin treatment group (10 mg/kg, once every 4 days), and the combination treatment group of cisplatin (3 mg/kg, once every 4 days) and remodelin (10 mg/kg, once every 4 days). Each group consisted of 7 mice. The tumor sizes of the mice were measured every 4 days. The tumor volume was calculated using the formula 0.5 × L × W² (where L = the long diameter of the tumor and W = the short diameter of the tumor). After six weeks of treatments, the mice were humanely euthanized, and the tumors were excised for subsequent analysis. For the rescue experiment of DUSP1 in nude mice, 5×10⁶ BGC-823/CR control cells, BGC-823/CR cells with NAT10 knockdown, BGC-823/CR cells with DUSP1 overexpression, and BGC-823/CR cells with both NAT10 knockdown and DUSP1 overexpression were also subcutaneously injected into the mice and treated according to the method described above. The tumors were photographed and weighed, and then fixed for terminal deoxynucleotidyl transferase dUTP nick end labeling (TUNEL) staining according to the manufacturer’s protocol (Cat# E-CK-A320, Elabscience).

For the immunotherapy experiment on C57BL/6 mice, 2×10^7^ mouse tumor cells MFC [59], MFC cells with stable overexpression of NAT10, the cisplatin-resistant MFC control cells (MFC/CR) and MFC/CR cells with stable knockdown of NAT10 were suspended in 100 μl of SPSS respectively and were injected into the right flanks of the C57 mice to allow tumor formation. Then the mice were injected intraperitoneally with anti-PD-1 monoclonal antibody or isotype IgG antibody (100 μg for each mouse, once every 3days) with or without remodelin (10 mg/kg, once every 3days). The mice were euthanized humanely at the indicated time point after being treated with 2 weeks and the tumors were harvested for subsequent analysis. The animal experiments were performed according to the guidelines of the Declaration of Helsinki and approved by the Experimental Animal Ethical Committee of Shandong University (ECSBMSSDU2023-2-15).

### Tumor sample digestion and flow cytometry analysis

The subcutaneous tumors of the mice were excised, digested, and a single-cell suspension was prepared using a Tumor Dissociation Kit (Cat# 130-096-730, Miltenyi Biotec). Then, the samples were filtered through a 70 μm cell strainer and subsequently stained with fluorescently labeled antibodies (anti-CD3, anti-CD8a, anti-TIM3, and anti-GzmB; BioLegend). Quantitative analysis was then performed using a flow cytometer (Beckman, CytoFLEX). The detailed information about the antibodies is included in Suppl. Table 5.

### Multiplex immunofluorescence staining

Multiplex immunofluorescence staining of tumor sample was performed using a Four-color Fluorescence kit (Recordbio Biological Technology, Shanghai, China) based on the tyramide signal amplification (TSA) technology according to the manufacture’s instruction. Briefly, paraffin sections were first deparaffinized, rehydrated, and subjected to antigen retrieval.After blocking with BSA, the sections were incubated sequentially with the primary antibody and an HRP-conjugated secondary antibody, with thorough washes performed after each incubation step. Tyramide signal amplification was subsequently conducted using a fluorophore-labeled substrate. Finally, nuclei were counterstained with DAPI, and the sections were mounted and imaged using an Olympus Fluorescence Microscope.

### Acetylation site prediction

The conserved acetylation sites of DUSP1, PD-L1 and FOSB mRNA were predicted by PACES tools (http://rnanut.net/paces/).

### Statistical analysis

Each experiment was performed at least 3 times. All data were presented as mean ± S.D. The differences between two groups were assessed using a two-tailed unpaired Student’s t-test. One-way ANOVA was used to compare the means of three or more experimental groups. Statistical analyses were performed using GraphPad Prism 8.0. 2. Statistical significance was denoted by* (*p* < 0.05), ** (*p* < 0.01), *** (*p* < 0.001), **** (*p* < 0.0001) and ns (no significant) in the figures.

## Supplementary information

Supporting information

original western blots

## Data Availability

The data that support the findings of this study are available from the corresponding author upon reasonable request.
