# Supplementary material for: NAT10 promotes cisplatin resistance and immune escape by increasing the expression of DUSP1 and PD-L1 in gastric cancer
Source: Cell Death Discov. 2026 Apr 10;12:237. doi: 10.1038/s41420-026-03107-w (PMC13187021; doi:10.1038/s41420-026-03107-w)
Supplement: Supplementary file 1 — Supporting information [file 41420_2026_3107_MOESM1_ESM.docx]

**Supplementary figures**

**NAT10 Promotes Cisplatin Resistance and Immune Escape by Increasing the Expression of DUSP1 and PD-L1 in Gastric Cancer**

*Lilin Qian^1^, Wenrong Gao^2^, Xinyi Wang^2^, Shuqi Cui^1^, Xiaoqi Han^1^, Xia Xu^1^, Jihui Jia^2^, Zhifang Liu^1^**


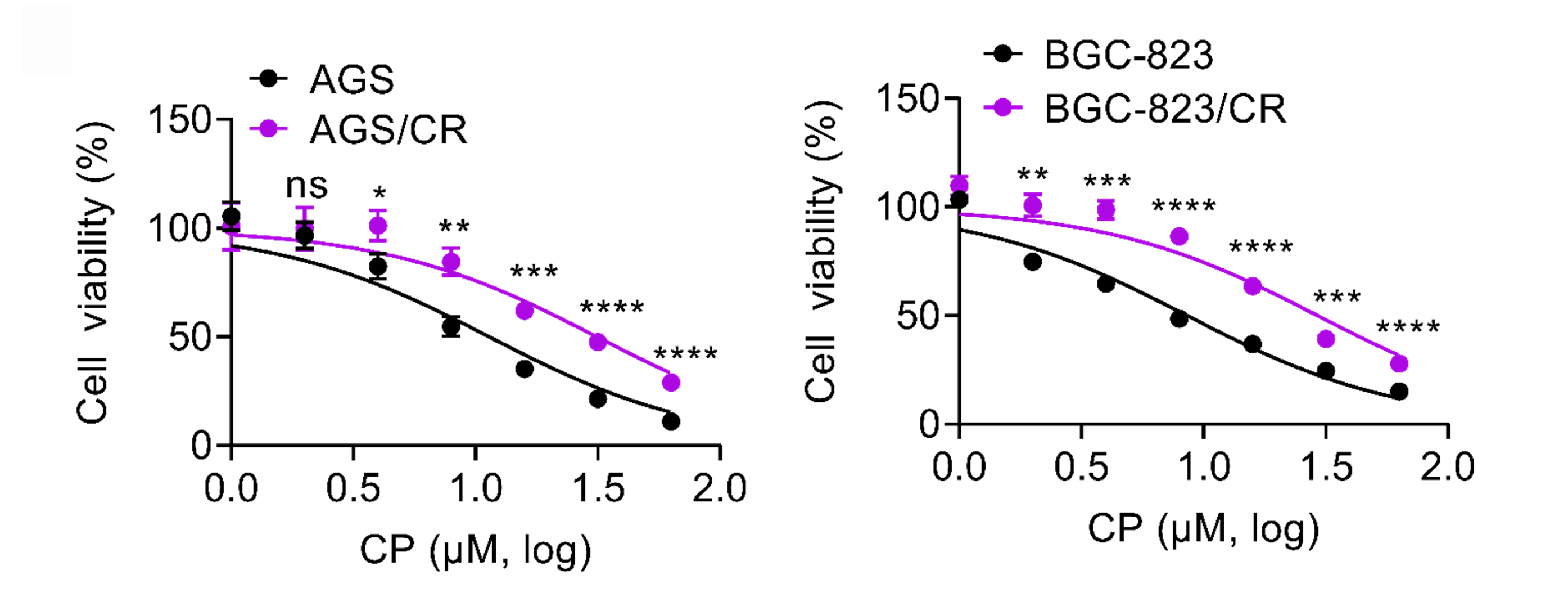


***Sup. Fig. 1****.* ***T*he half-maximal inhibitory concentration (IC_50_) of cisplatin in resistant cells (AGS/CR and BGC-823/CR) was significantly higher than that in the parental cells (AGS and BGC-823),as determined by CCK-8 assay.**
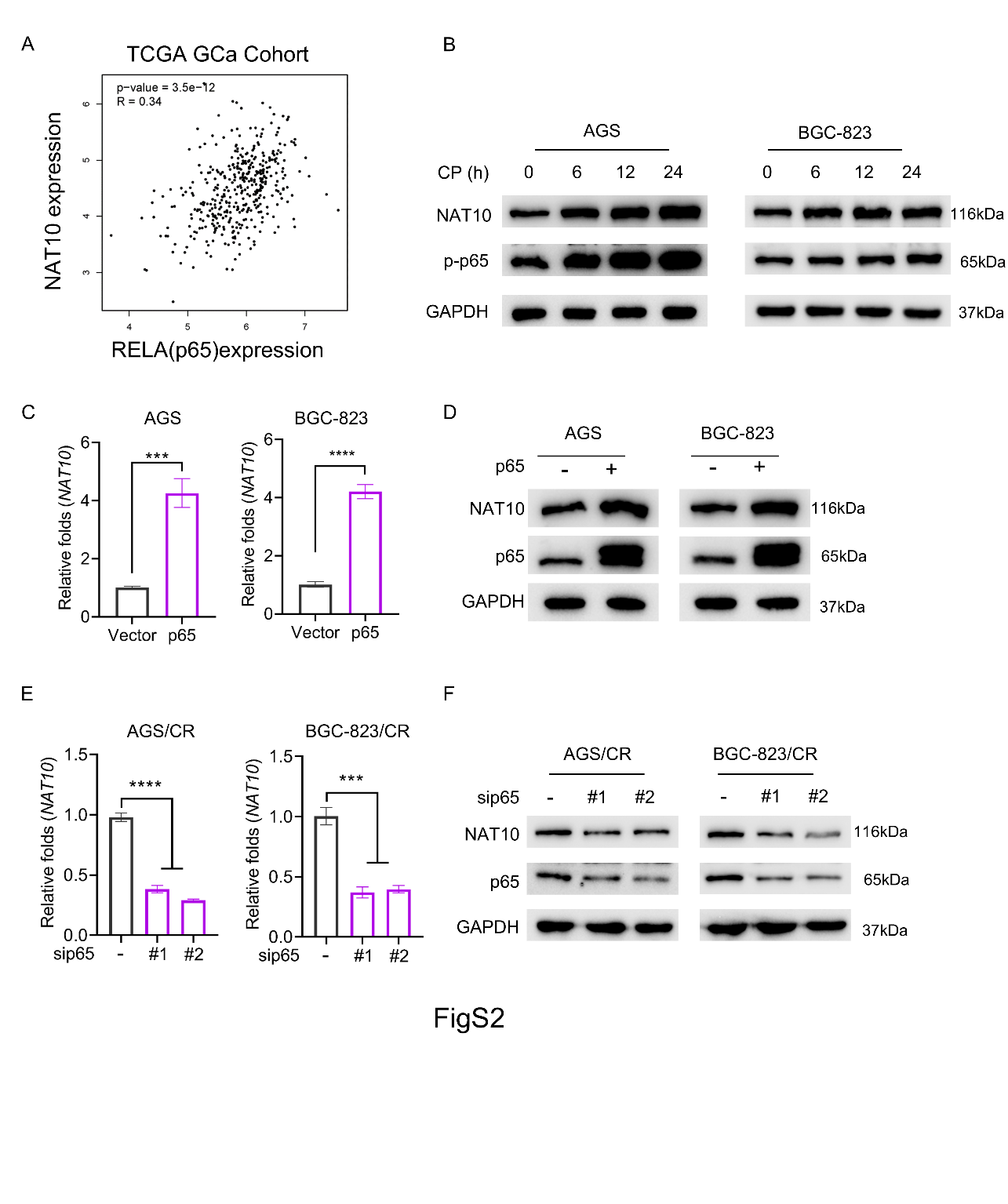


***Sup.Fig.2. Cisplatin promotes NAT10 transcription via p65 in gastric cancer***

1. Pearson correlation analysis of the relationship between RELA (p65) expression and NAT10 expression.
2. Expression levels of NAT10 and p-p65 were analyzed by Western blot at different time points following cisplatin treatment.
3. **and** **(D)** NAT10 expression was analyzed by qRT-PCR and Western blot following p65 overexpression.

(**E**) and (**F**) NAT10 expression was analyzed by qRT-PCR and Western blot following p65 knockdown.


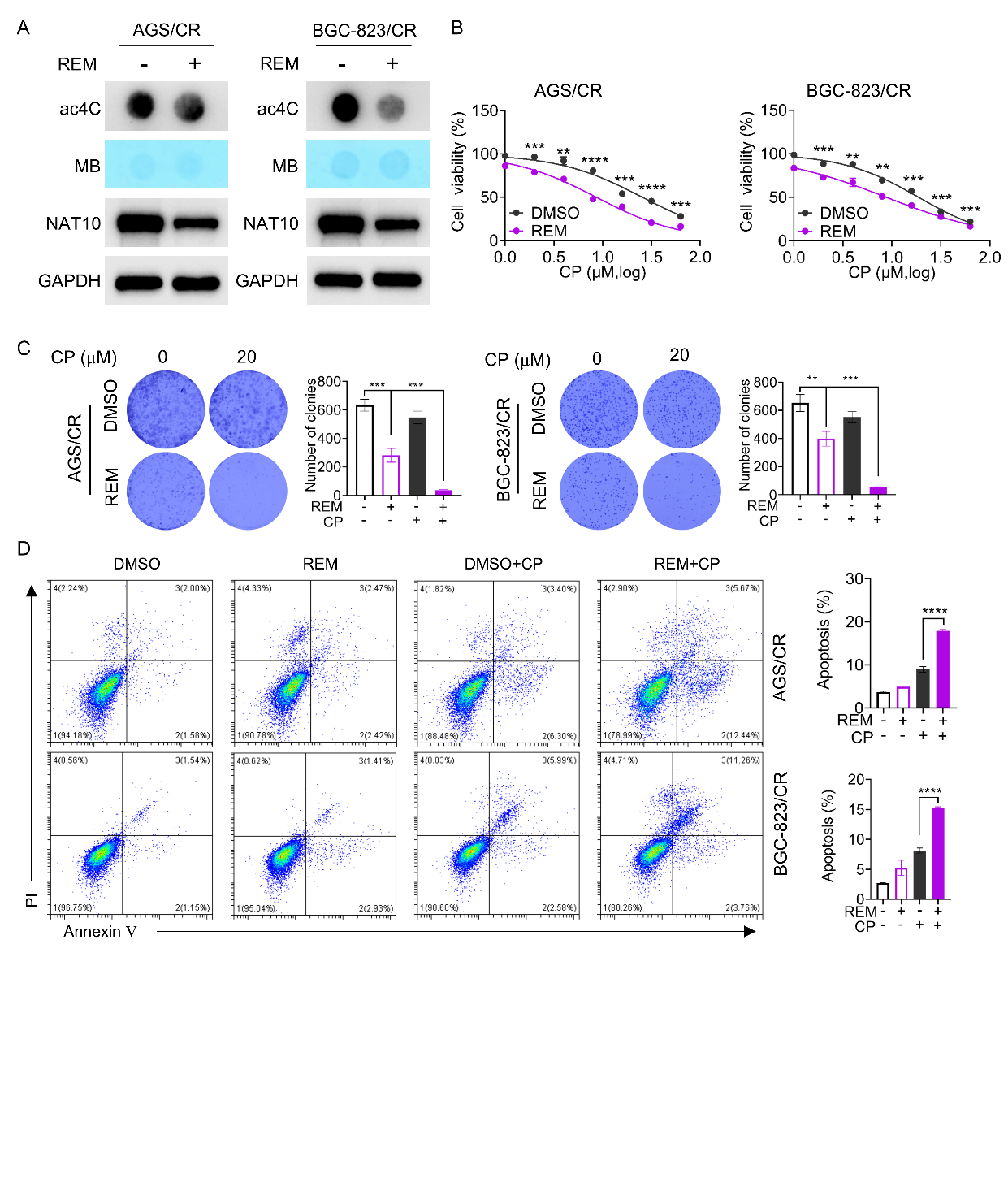


***Sup. Fig. 3.*** ***Remodelin enhances the cisplatin sensitivity of gastric cancer cells in vitro****.*

1. Total RNA ac4C modification levels and NAT10 expression were detected by RNA dot blot and Western blot, respectively, in remodelin-treated AGS/CR and BGC-823/CR cells.

**(B)** Cell viability was determined by CCK-8 assay in AGS/CR and BGC-823/CR cells treated with cisplatin alone or in combination with remodelin.

**(C)** Colony formation assay in AGS/CR and BGC-823/CR cells treated with cisplatin alone or in combination with remodelin.

**(D)** Flow cytometry was used to detect cell apoptosis in cisplatin-resistant cells treated with cisplatin alone or in combination with remodelin.


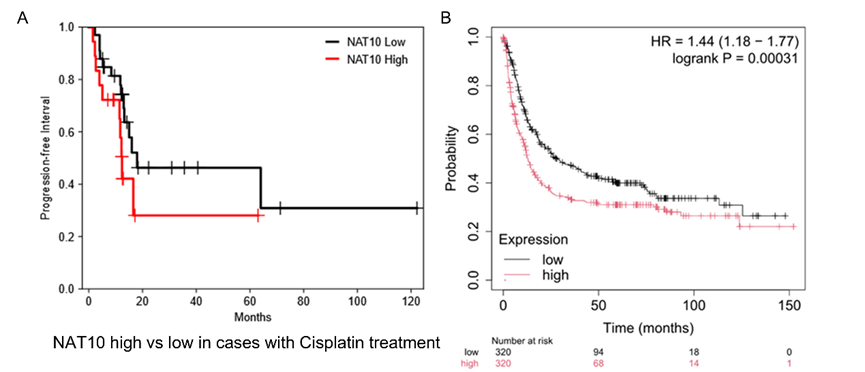


***Sup. Fig. 4* （A）** Data from the GEPIA3 database (https://gepia3.bioinfoliu.com) indicated that high NAT10 expression corresponded to shorter progression-free survival (PFS) in cisplatin-treated gastric cancer patients.

**(B)** High NAT10 expression was significantly associated with a shorter Progression-Free Survival in the cohort of gastric cancer patients. (https://www.kmplot.com/analysis)***
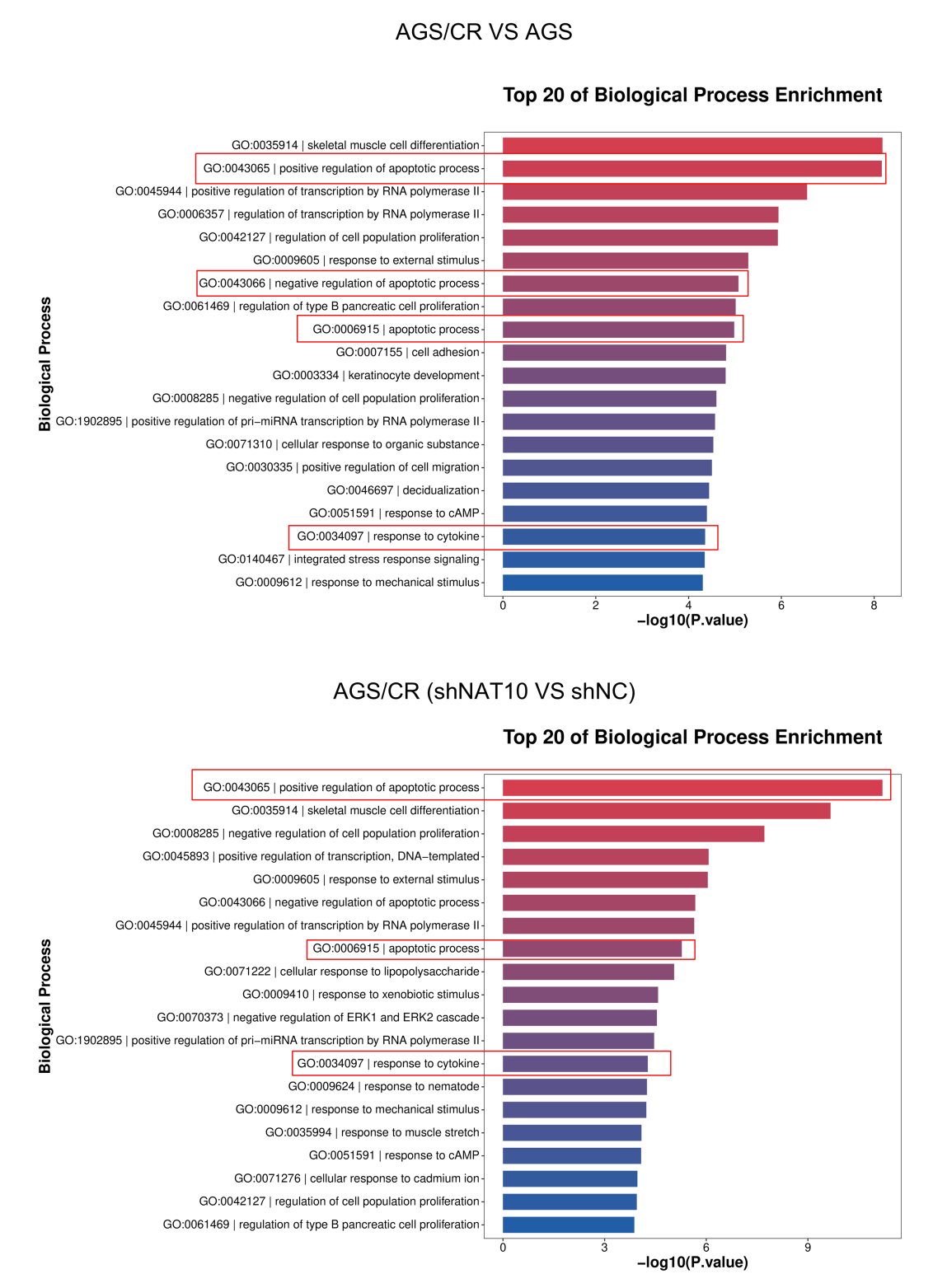
***

***Sup. Fig. 5.***  ***Biological Process GO pathway enrichment analysis for the differentially expressed gene.***


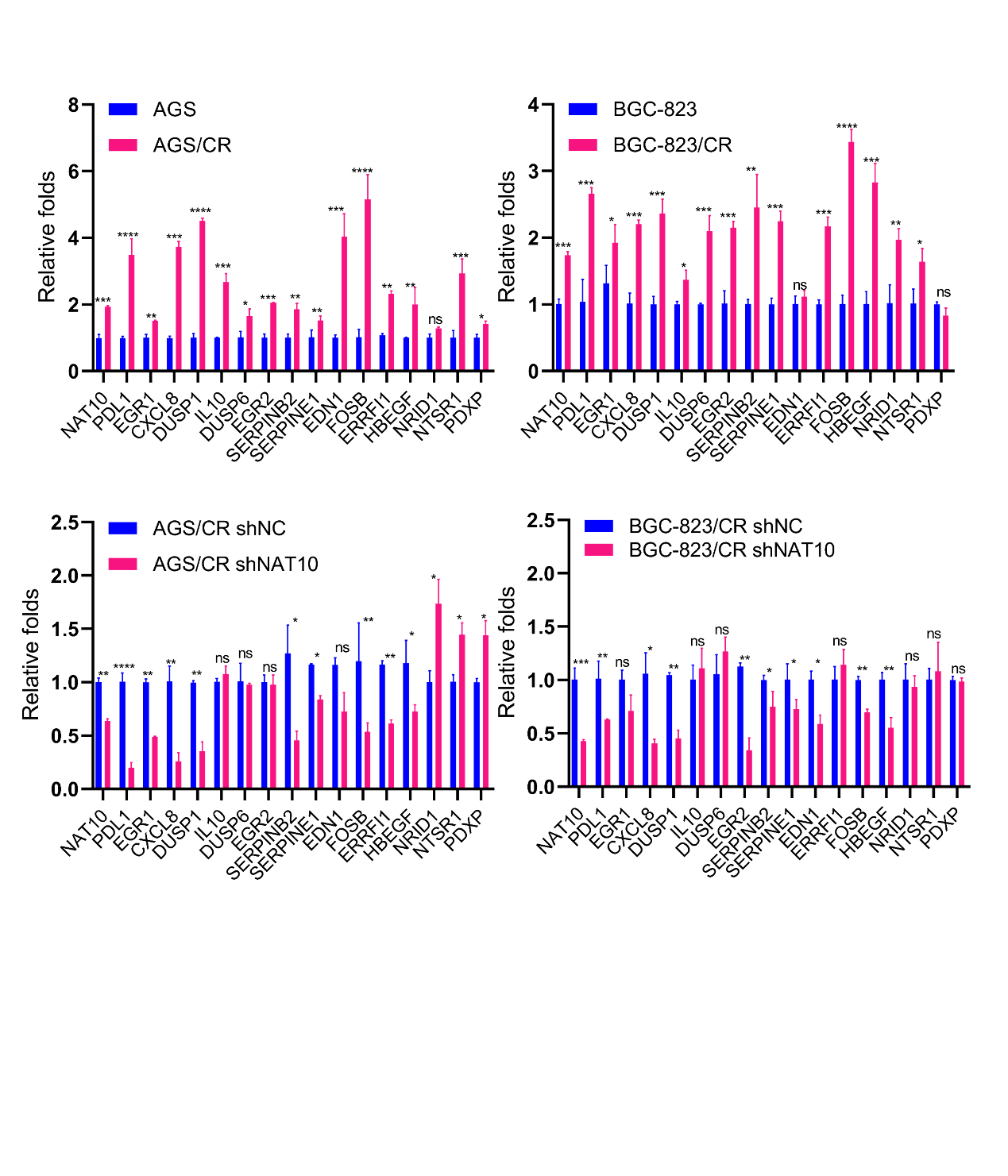


***Sup. Fig. 6. RT-qPCR was used to verify the differential expressed genes from transcriptome sequencing results.***

**
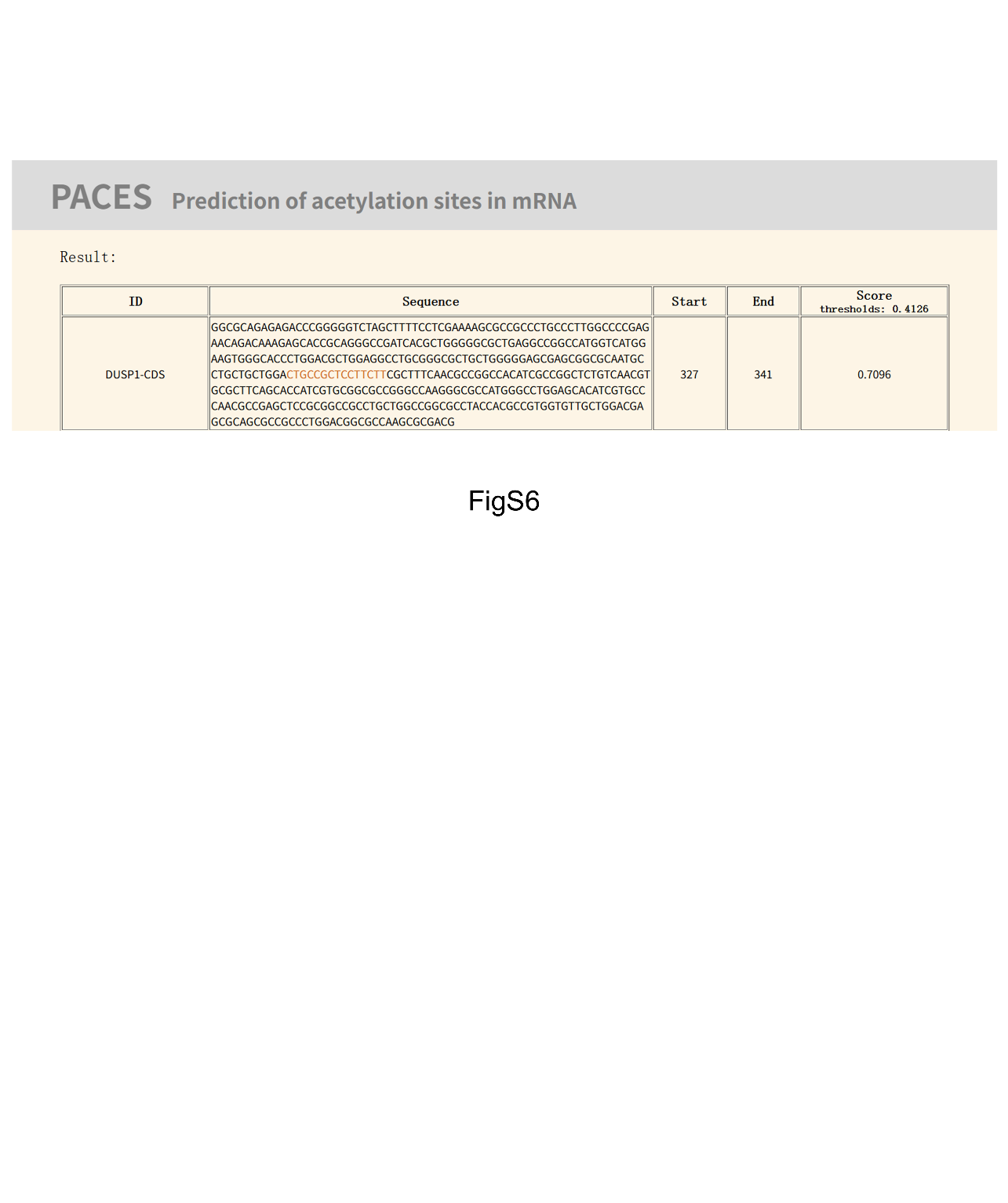
**

***Sup. Fig. 7.*** ***PACES tool (http://rnanut.net/paces/) was used to predict the conserved acetylation sites in the DUSP1 mRNA CDS.***

**
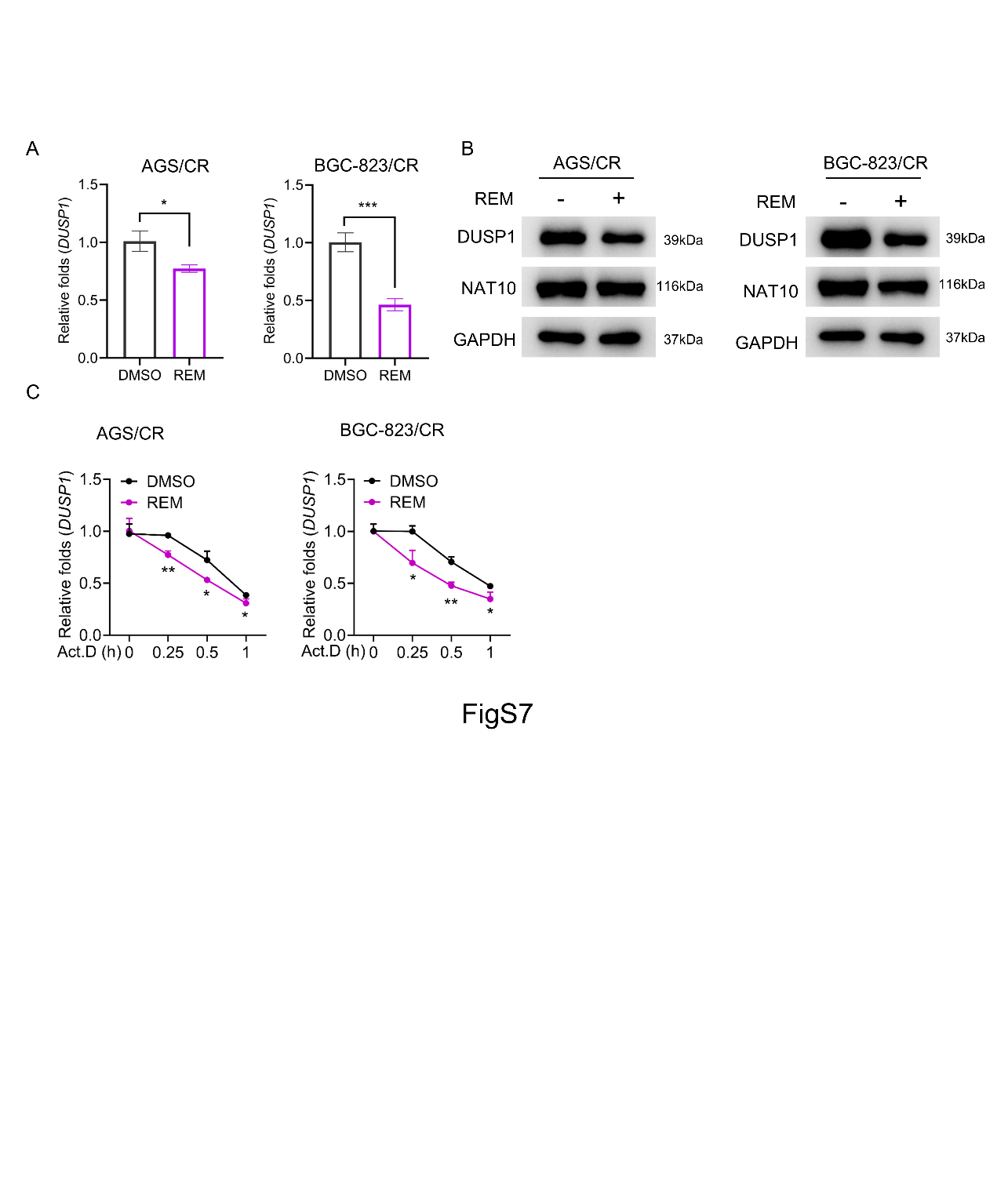
**

***Sup. Fig. 8.*** ***Remodelin inhibits DUSP1 mRNA stability and protein expression level.***

**(A) and (B)** The expression level of DUSP1 was detected by RT-qPCR and Western blot in AGS/CR or BGC-823/CR cells treated with remodelin.

**(C)** The half-life of DUSP1 mRNA was detected in AGS/CR or BGC-823/CR cells treated with remoldelin.


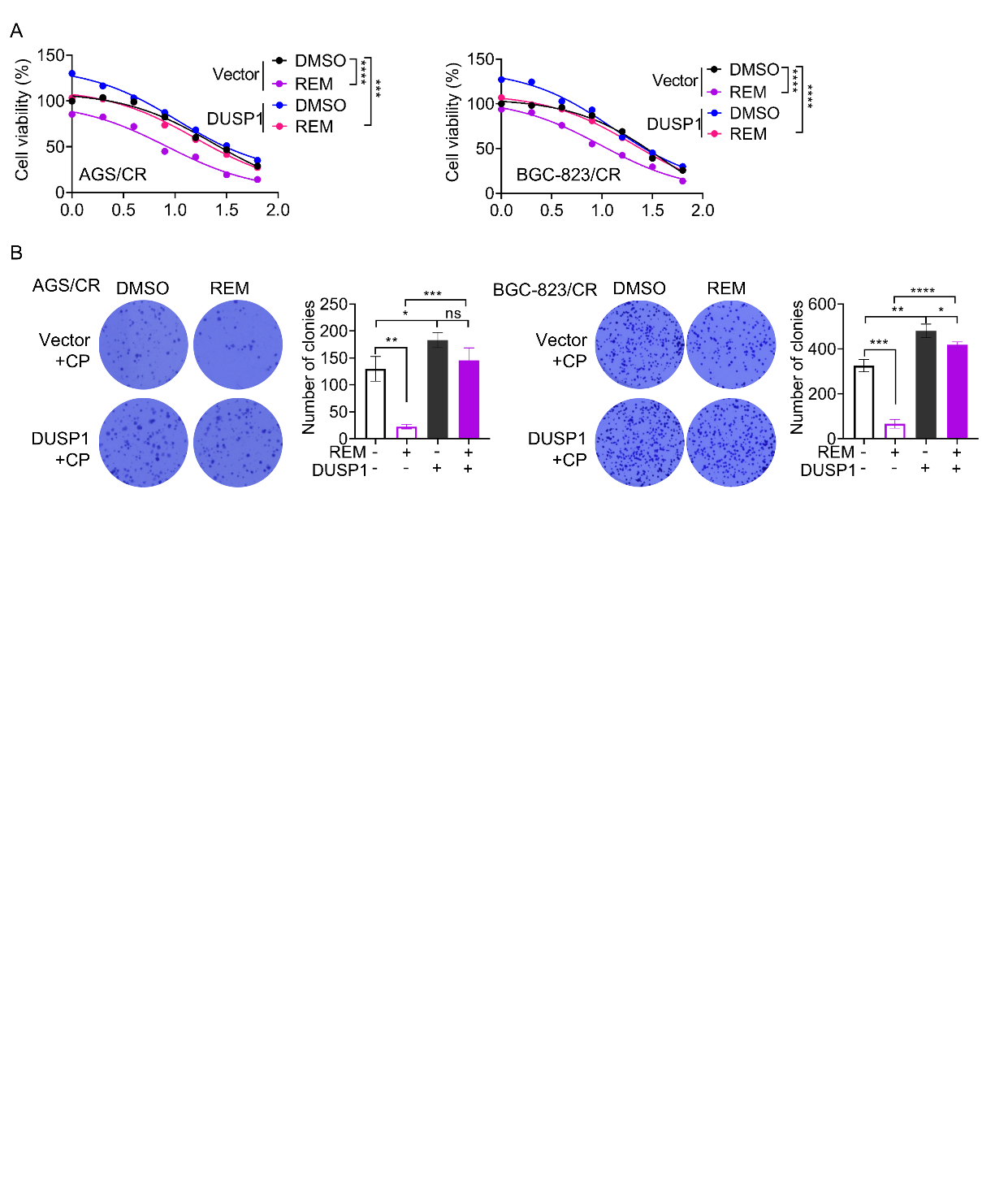


***Sup. Fig. 9. Overexpression of DUSP1 attenuated the remodelin-induced increase in the sensitivity of gastric cancer cells to cisplatin.***

**(A)** Cell viability was assessed via CCK-8 assay in AGS and BGC-823 cells transfected with a DUSP1 expression vector, followed by treatment with cisplatin combined with either DMSO or remodelin.

**(B)** Colony formation ability was evaluated using the colony formation assay in AGS and BGC-823 cells transfected with a DUSP1 expression vector, followed by treatment with cisplatin combined with either DMSO or remodelin.


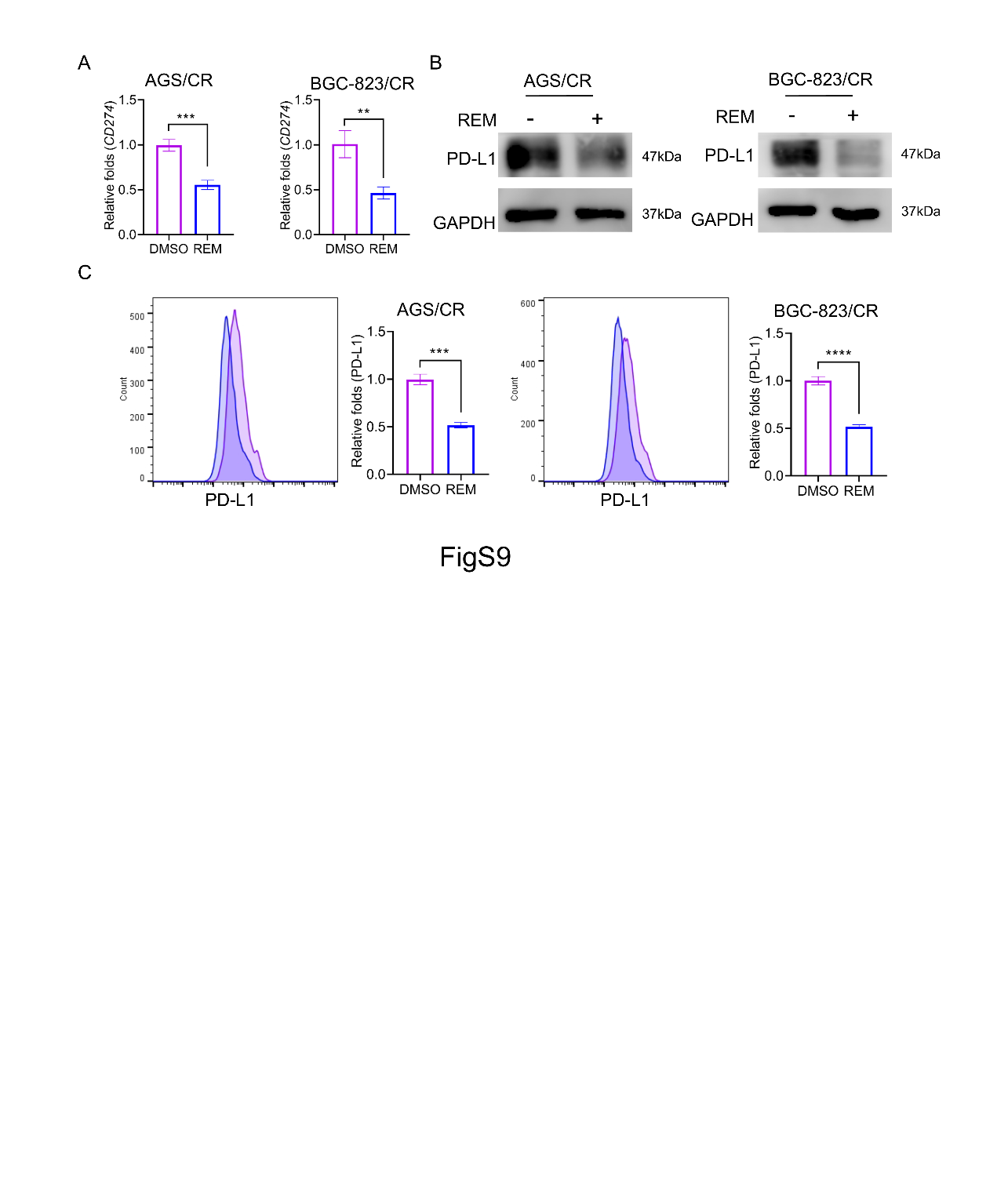


***Sup. Fig. 10. Remodelin inhibits PD-L1 expression in cisplatin-resistant cells.***

**(A)** Expression of PD-L1 mRNA (CD274) was examined by RT- qPCR in cisplatin-resistant cells treated with remodelin.

**(B)** Expression of PD-L1 was examined by Western blot in cisplatin-resistant cells treated with remodelin.

**(C)** Expression of PD-L1 on the cell surface was examined by flow cytometry in cisplatin-resistant cells treated with remodelin.


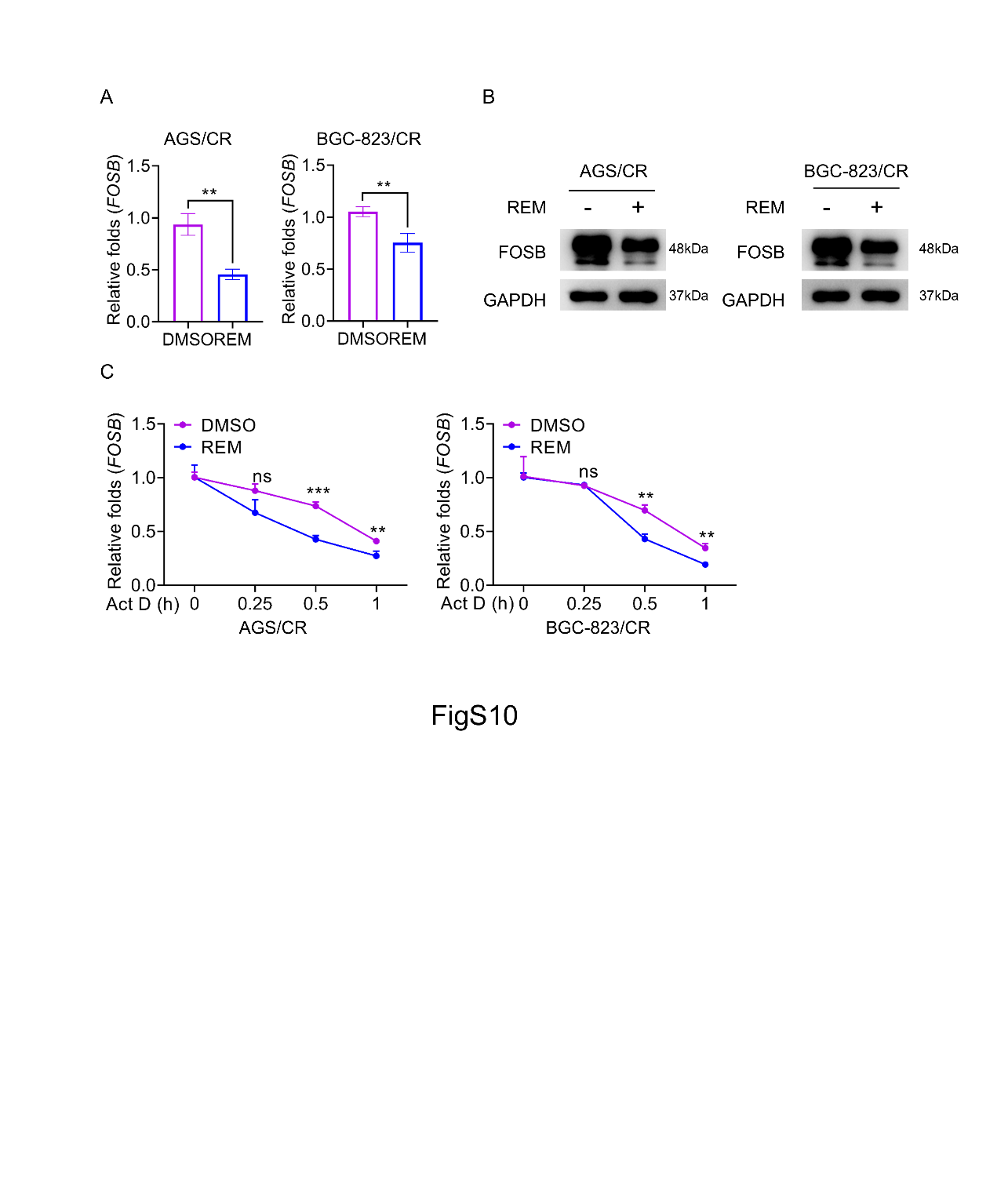


***Sup. Fig. 11. Remodelin inhibits FOSB mRNA stability and expression.***

**(A) and (B)** The expression level of FOSB in AGS/CR or BGC-823/CR cells treated with remodelin was examined by RT-qPCR and Western blot, respectively.

**(C)** The stability of FOSB was detected in AGS/CR or BGC-823/CR cells treated with remoldelin.


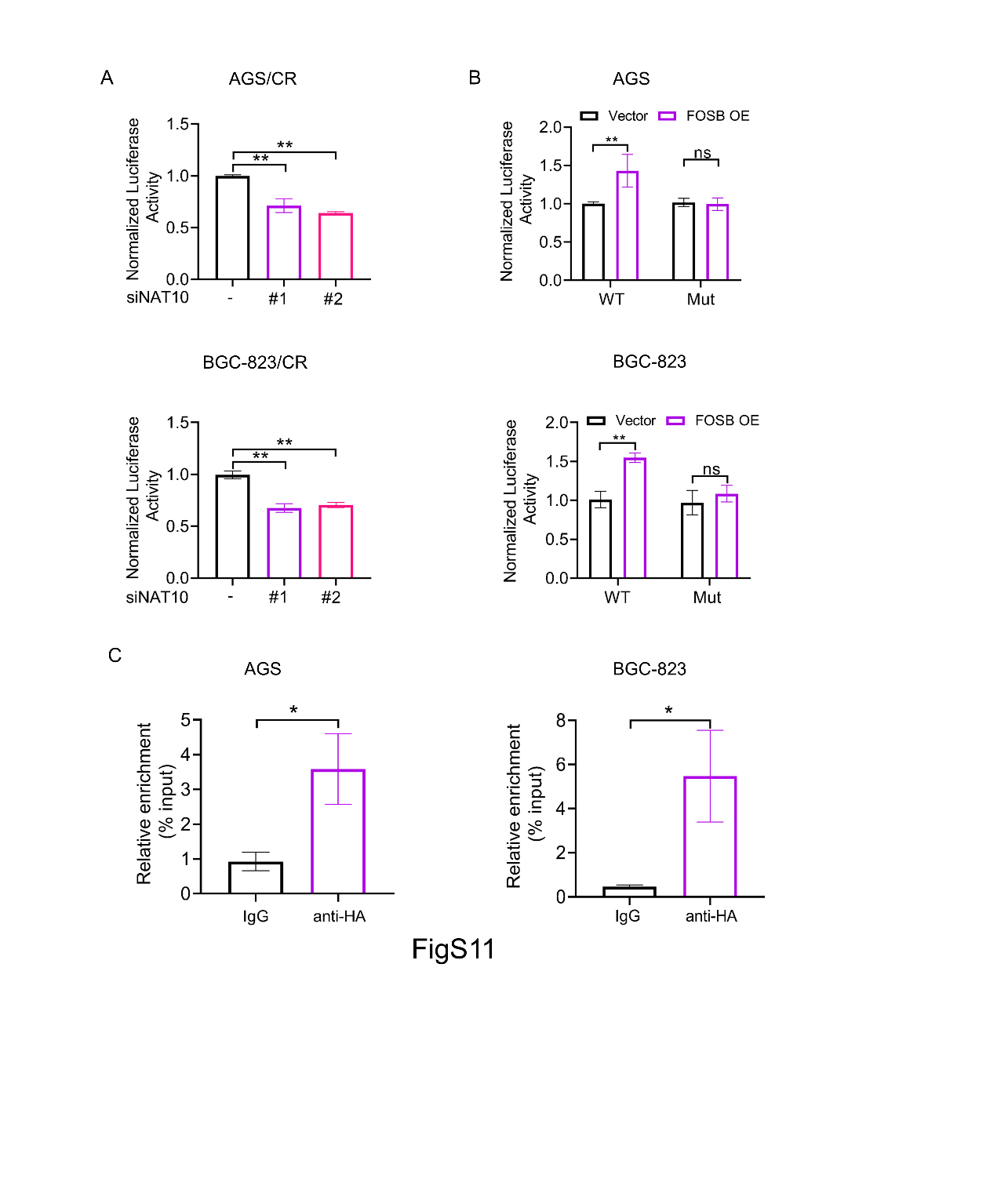


***Sup. Fig. 12. FOSB binds to the promoter of PD-L1 and regulates its expression***

**(A)** Detection of dual-luciferase reporter gene assay in AGS/CR or BGC/CR cells transfected with the pGL3-CD274 promoter vector, pRL-TK vector, and either control siRNA or FOSB siRNA.

**(B)** Dual-luciferase reporter gene activity in AGS or BGC-823 cells transfected with the CD274 wild-type or mutant (Site -288~-297) promoter luciferase vector, pRL-TK vector, and either the empty vector or FOSB expression vector.

**(C)** ChIP-qPCR was performed to detect the binding of FOSB to the CD274 promoter in AGS and BGC-823 cells transfected with the HA-tagged FOSB expression vector.


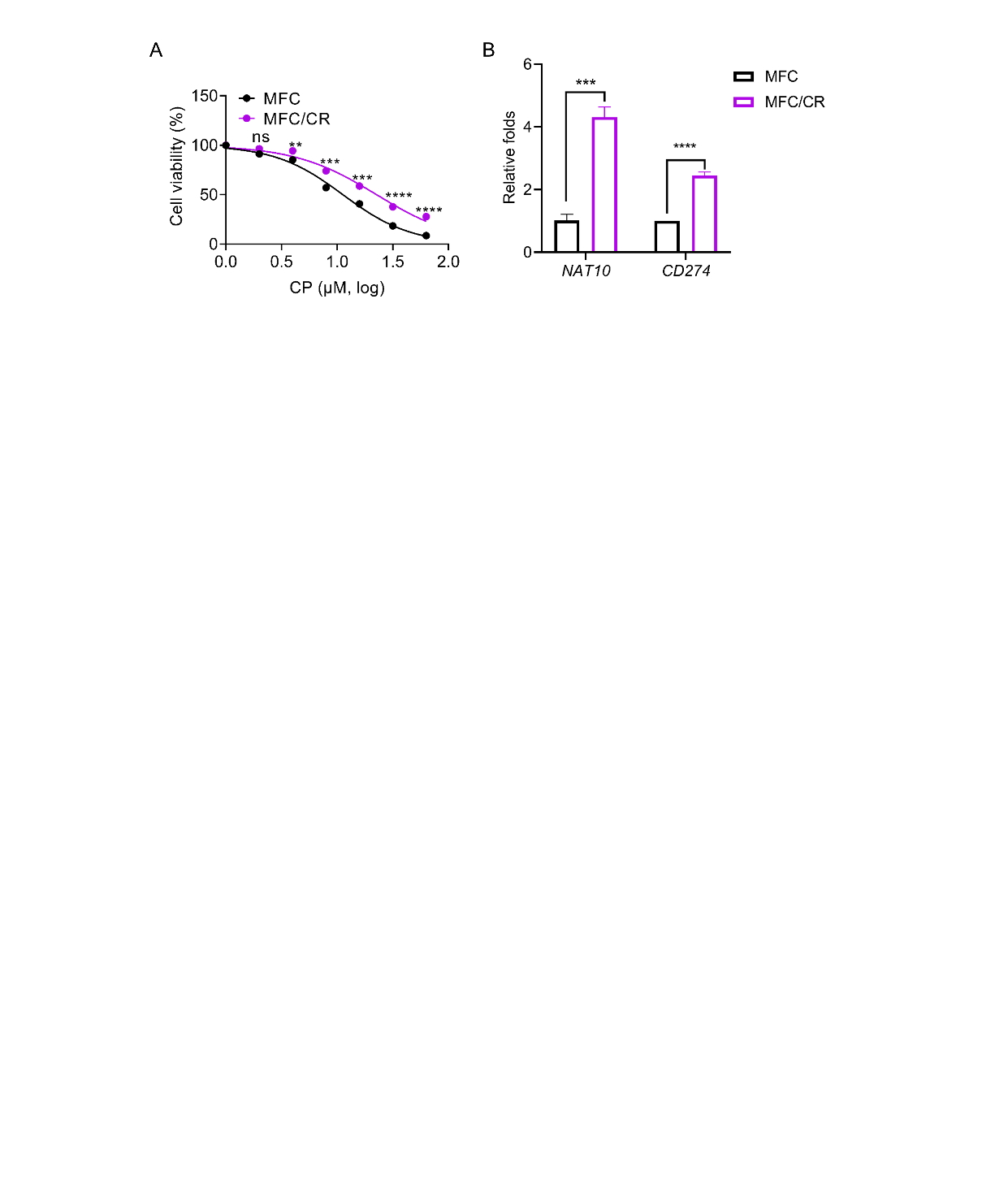


***Sup. Fig. 13. Verification of the cisplatin-resistant MFC cell line***.

**(A)** Cell viability ability of parental cells (MFC) and cisplatin-resistant cells (MFC/CR) was determined by CCK-8 assays.

**(B)** Expression of CD274 was examined by RT-qPCR in MFC and MFC/CR.


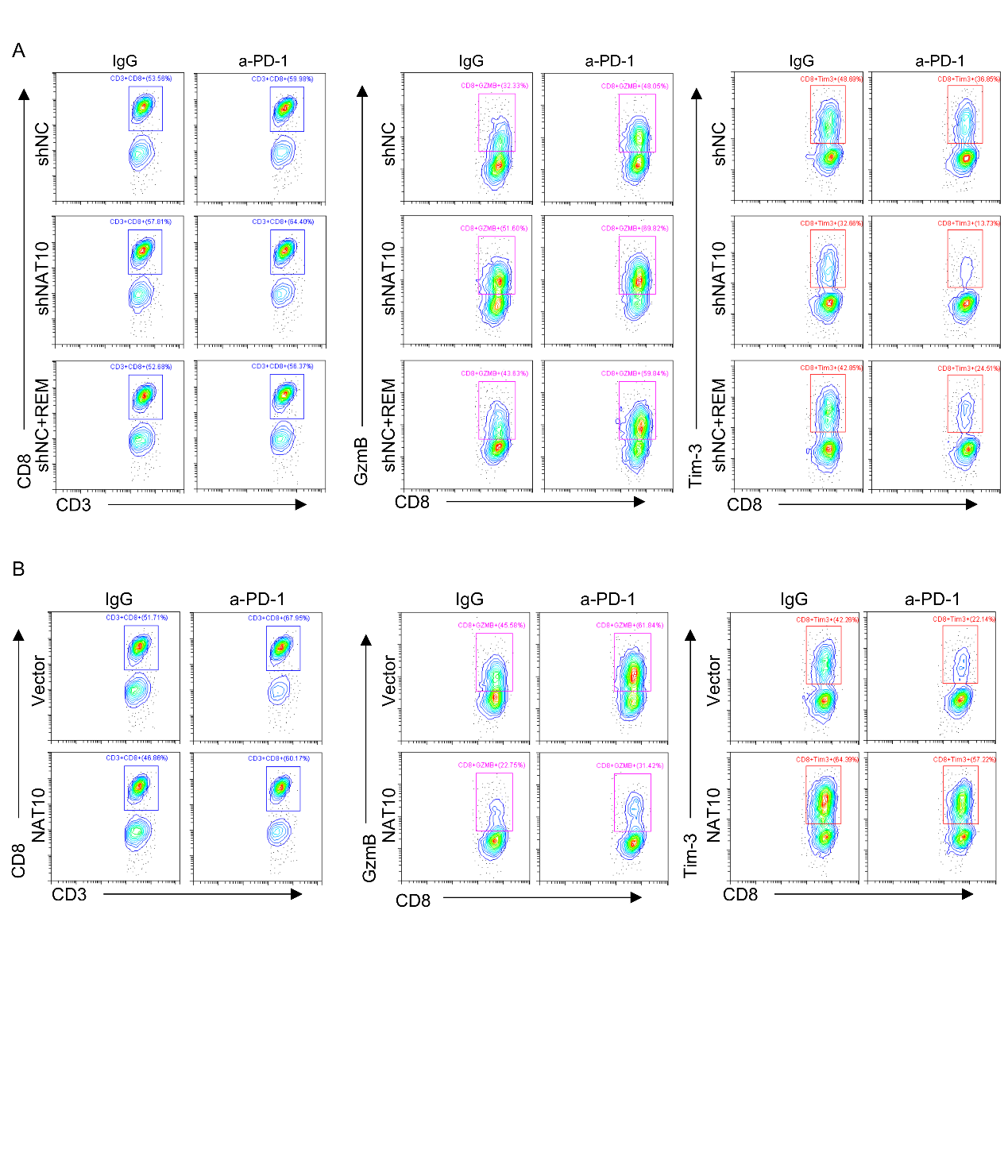


***Sup. Fig. 14. T cells infiltrating the tumor was analyzed using flow cytometry***

**(A) and (B)** The percentages of CD3^+^ CD8^+^, CD8^+^ GzmB^+^ and CD8^+^ Tim3^+^ T cells infiltrating the tumor were analyzed using flow cytometry in different treatment groups.

***Supplementary tables***

**Table 1. Clinical Information of Samples**

| Pathological Type | Degree of  Differentiation | T Stage | Lymph Node Metastasis | Tumor size  (cm) |
| --- | --- | --- | --- | --- |
| Adenocarcinoma | Poorly | T4 | N1 | 3×4×1 |
| Adenocarcinoma | Poorly | T2 | N1 | 3×3×1 |
| Adenocarcinoma | Poorly | T3 | N2 | 5×5×2 |
| Adenocarcinoma | Poorly | T4 | N1 | 8×5×5 |
| Adenocarcinoma | Poorly | T3 | N1 | 4×4×3 |
| Adenocarcinoma | Poorly | T4 | N1 | 5×4×3 |
| Adenocarcinoma | Poorly | T4 | N2 | 4×3×1 |
| Adenocarcinoma | Poorly | T4 | N3 | 3×2×1 |
| Adenocarcinoma | Moderately | T3 | N3 | 2×2×1 |
| Adenocarcinoma | Moderately | T1 | N0 | 3×2×0.3 |
| Adenocarcinoma | Well | T1 | N0 | 3×2×1 |
| Adenocarcinoma | Well | T1 | N0 | 2×1.5×1 |

| **Table 2. siRNA and shRNA sequence used in this study** | | | |
| --- | --- | --- | --- |
| **siRNA** | **Sequences** | | |
| si-Ctrl | | UUCUCCGAACGUGUCACGUTT | |
| si-H-NAT10-1 | | GCACCACUGCUGAGAAUAATT | |
| si-H-NAT10-2 | | CCAUCUCUCGCAUCUAUUUTT | |
| si-DUSP1-1 | | TAGCGTCAAGACATTTGCTGA | |
| si-DUSP1-2 | | CACGAACAGTGCGCTGAGCTA | |
| si-FOSB-1 | | CCGUUGUUAACCCUUCGUATT | |
| si-FOSB-2 | | CUCAAUAUCUGUCUUCGGUTT | |
| **shRNA** | | | **Sequences** |
| sh-Ctrl | | | TTCTCCGAACGTGTCACGT |
| sh-H-NAT10 | | | GCACCACTGCTGAGAATAA |
| sh-Ctrl | | | TTCTCCGAACGTGTCACGTTT |
| sh-M-NAT10 | | | GCTTCTCAAGTTCTGGAAATT |

**Table 3 Oligos sequences for plasmids construction**

| Name | Sequences 5’-3’ | Note |
| --- | --- | --- |
| NAT10 mut  (site K290A) | F:ACGGGGAGCATCTGCAGCCCTGGGATTGGCGA | Site-Directed Mutagenesis |
| NAT10 mut  (site K290A) | R:CTGCAGATGATCCCCGTCCTCGAGCAGCTGTG | Site-Directed Mutagenesis |
| NAT10 mut  (site G641E) | F:TATGAGAGCCGTGCTCTGCAGCTGCTGCAGAT | Site-Directed Mutagenesis |
| NAT10 mut  (site G641E) | R:AGAGCACGGCTctcATAGCCCATCCCTTGATAATCTGG | Site-Directed Mutagenesis |
| DUSP1-CDS-mut1 | F:GAATGCCGCTCCTTCTTCGCTTTCAACGCCGG | Site-Directed Mutagenesis |
| DUSP1-CDS-mut1 | R:AAGAAGGAGCGGCATTCCAGCAGCAGGCATTGC | Site-Directed Mutagenesis |
| DUSP1-CDS-mut2 | F：TGGACTGAAGCTCCTTCTTCGCTTTCAACG | Site-Directed Mutagenesis |
| DUSP1-CDS-mut2 | R:AGAAGGAGCTTCAGTCCAGCAGCAGGCATTG | Site-Directed Mutagenesis |
| DUSP1-CDS-mut3 | F:GACTGCCGATCCTTCTTCGCTTTCAACGCCGG | Site-Directed Mutagenesis |
| DUSP1-CDS-mut3 | R:AAGAAGGATCGGCAGTCCAGCAGCAGGCATTG | Site-Directed Mutagenesis |
| DUSP1-CDS | F:AGTGAGGGAGCTCATAGGCCATGGTCATGGAAGTGGGCAC | In-Fusion cloning |
| DUSP1-CDS | R:ATCACGCACGCGTCTATGCCTTACTCGAGGCAGCTGGGAG | In-Fusion cloning |
| pmirGLO vector | F: GGCATAGACGCGTGCGTG | In-Fusion cloning |
| pmirGLO vector | R: GGCCTATGAGCTCCCTCACTAG | In-Fusion cloning |
| T7-DUSP1-Sense | F: CGAAATTAATACGACTCACTATAGG | In-Fusion cloning |
| T7-DUSP1-Sense | R: TCACTCGAGGCAGCTGGG | In-Fusion cloning |
| pGL3-CD274-MUT-F | F:GATGGCCTGGATGATCTTTTTAAACTGGGGATGGGTATTT | In-Fusion cloning |
| pGL3-CD274-MUT-R | R: AAGATCATCCAGGCCATCTAGGAGGATATGATT | In-Fusion cloning |

**Table 4 Primers used in this study**

| **Primer Name** | **Sequence 5'-3'** | |
| --- | --- | --- |
| H-β-actin Forward | CATGTACGTTGCTATCCAGGC | |
| H-β-actin Reverse | | CTCCTTAATGTCACGCACGAT |
| H-NAT10 Forward | ATAGCAGCCACAAACATTCGC | |
| H-NAT10 Reverse | ACACACATGCCGAAGGTATTG | |
| H-CD274 Forward | GGTAAGACCACCACCACCAAT | |
| H-CD274 Reverse | TGATTCTCAGTGTGCTGGTCAC | |
| DUSP1 Forward | CATGGTCATGGAAGTGGG | |
| DUSP1 Reverse | GCGTTGAAAGCGAAGAAG | |
| CXCL8 Forward | ACTGAGAGTGATTGAGAGTGGAC | |
| CXCL8 Rerverse | AACCCTCTGCACCCAGTTTTC | |
| EGR1 Forward | GGTCAGTGGCCTAGTGAGC | |
| EGR1 Reverse | GTGCCGCTGAGTAAATGGGA | |
| IL10 Forward | GACTTTAAGGGTTACCTGGGTTG | |
| IL10 Reverse | TCACATGCGCCTTGATGTCTG | |
| DUSP6 Forward | GAACTGTGGTGTCTTGGTACATT | |
| DUSP6 Reverse | GTTCATCGACAGATTGAGCTTCT | |
| EGR2 Forward | TCTTCCCAATGATCCCAGACT | |
| EGR2 Reverse | TTACGGATTGTAGAGAGTGGAGT | |
| SERPINB2Forward | CAGCACCGAAGACCAGATGG | |
| SERPINB2 Reverse | CCTGCAAAATCGCATCAGGATAA | |
| SERPINE 1 Forward | AGTGGACTTTTCAGAGGTGGA | |
| SERPINE 1 Reverse | GCCGTTGAAGTAGAGGGCATT | |
| EDN1 Forward | AAGGCAACAGACCGTGAAAAT | |
| EDN1 Reverse | CGACCTGGTTTGTCTTAGGTG | |
| ERRFI1 Forward | GACCCACCGAAGATTAAGAAGG | |
| ERRFI1 Reverse | GGTCTAGGAGGTATGGGAACTCT | |
| FOSB Forward | GCTGCAAGATCCCCTACGAAG | |
| FOSB Reverse | ACGAAGAAGTGTACGAAGGGTT | |
| HBEGF Forward | ATCGTGGGGCTTCTCATGTTT | |
| HBEGF Reverse | TTAGTCATGCCCAACTTCACTTT | |
| NR1D1 Forward | TGGACTCCAACAACAACACAG | |
| NR1D1 Reverse | GATGGTGGGAAGTAGGTGGG | |
| NTSR1 Forward | AGCAGTGGACTCCGTTCCT | |
| NTSR1 Reverse | GTTGGCAGAGACGAGGTTGT | |
| PDXP Forward | CTGGAGACCGACATCCTCTTT | |
| PDXP Reverse | TTCTAGGCGGGAGACTCCTG | |
| CD274-CHIP-Forward | GATGGCCTGGATGATCTATG | |
| CD274-CHIP-Reverse | TTCCGTTTGCCTCATTGAT | |
| Mus-β-actin Forward | GGCTGTATTCCCCTCCATCG | |
| Mus-β-actin Reverse | CCAGTTGGTAACAATGCCATGT | |
| Mus-NAT10- Forward | GCGGCAGAGGTCTCTTTTTGT | |
| Mus-NAT10- Reverse | GTGACTGCTAAATCCCAGCTC | |
| Mus-CD274- Forward | TCAATGCCCCATACCGCAAA | |
| Mus-CD274- Reverse | TCTCTTCCCACTCACGGGTT | |

**Table 5. Antibodies used in this study**

| **Antibody Name** | **Source** | **Item number** |
| --- | --- | --- |
| NAT10 | Abcam | ab194297 |
| N4-acetylcytidine | Abcam | ab252215 |
| Cleaved-capase-3 | Abcam | ab32042 |
| β-actin | CST | 3700 |
| Ki67 | Servicebio | GB111499 |
| DUSP1 | Abways | CY6588 |
| GAPDH | Abways | ab0037 |
| Phospho-JNK1/2/3 | Abways | CY5541 |
| JNK1/2/3 | Abways | CY5490 |
| ERK | Abways | CY5490 |
| Phospho-ERK1/2 | Abways | CY5277 |
| PARP | Santa | sc-1562 |
| FOSB | HUBIO | ET1611-75 |
| PD-L1/CD274 | Proteintech | 66248-1-Ig |
| CD8a | Proteintech | 29896-1-AP |
| Granzyme B | Proteintech | 13588-1-AP |
| PE anti-human CD274 | BioLegend | 329706 |
| PerCP/Cyanine5.5 anti-mouse CD45 | BioLegend | 103132 |
| FITC anti-mouse CD3 | BioLegend | 100204 |
| APC anti-mouse CD8a | BioLegend | 100712 |
| PE/Cyanine7 anti-mouse CD366 (Tim-3) | BioLegend | 134010 |
| PE anti-human/mouse Granzyme B Recombinant | BioLegend | 396406 |
| InVivoPlus anti-mouse PD-1 | Bioxcell | BP0146 |
| HA | Proteintech | 51064-2-AP |
