# Supplementary figures and images for: NAT10 promotes cisplatin resistance and immune escape by increasing the expression of DUSP1 and PD-L1 in gastric cancer
Source: Cell Death Discov. 2026 Apr 10;12:237. doi: 10.1038/s41420-026-03107-w (PMC13187021; doi:10.1038/s41420-026-03107-w)

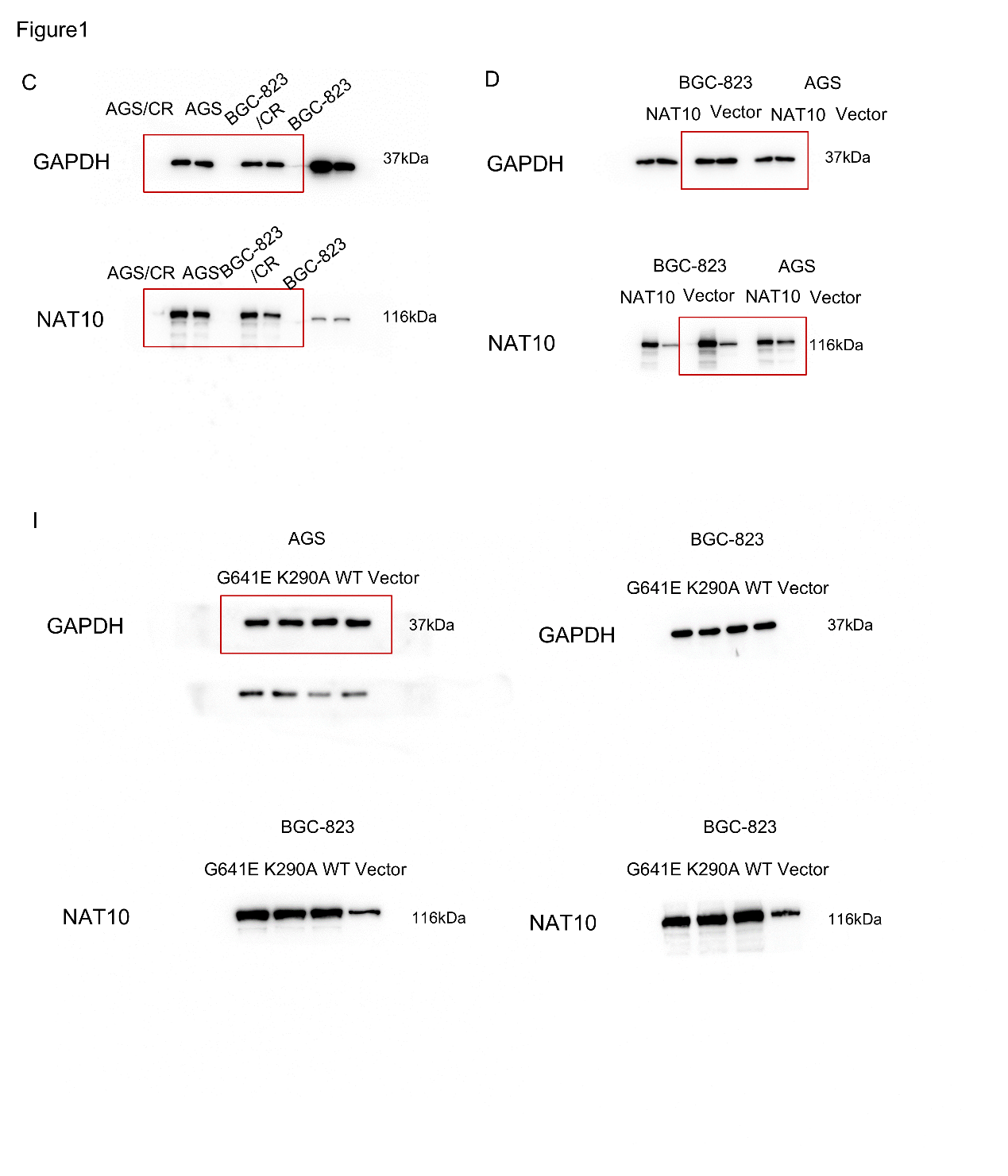

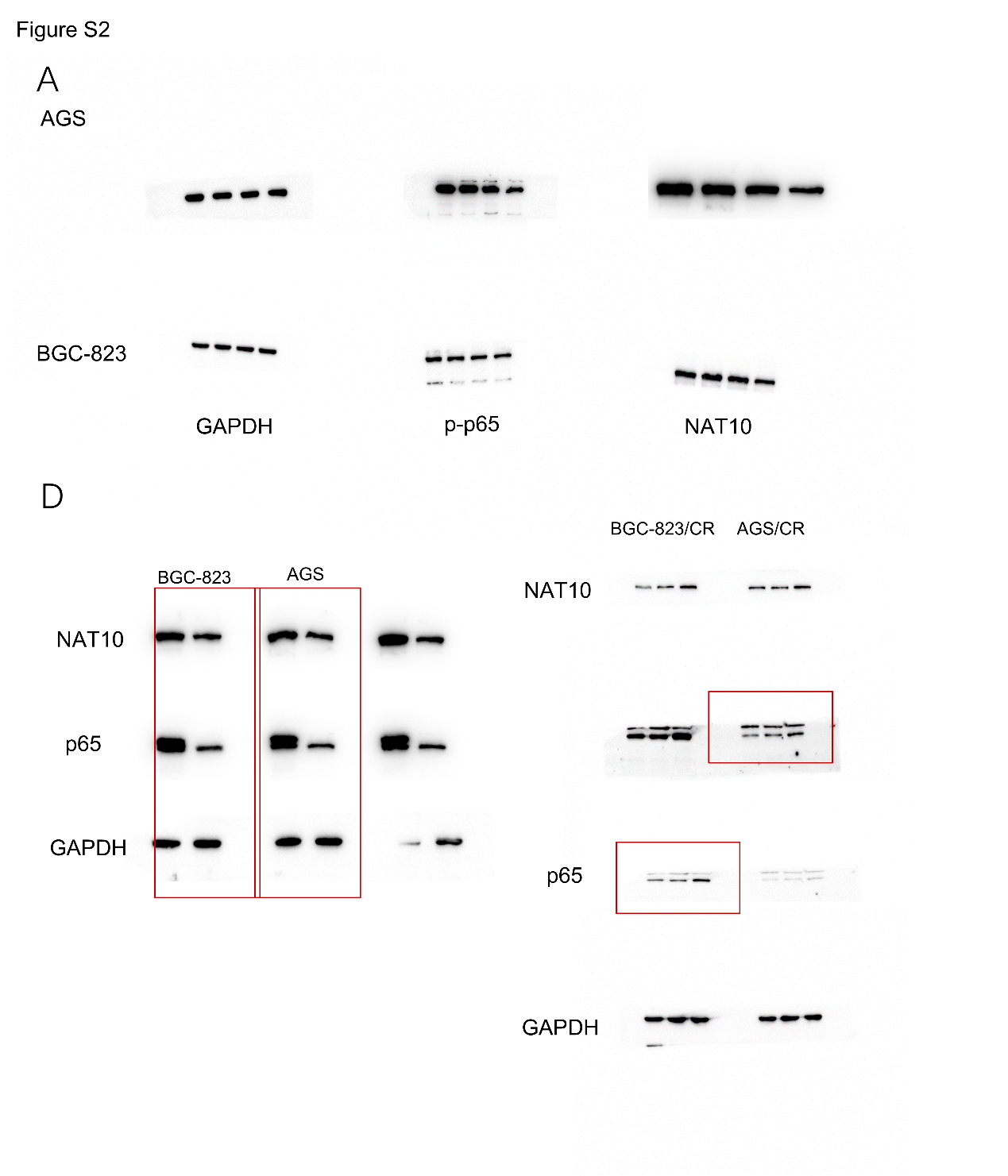

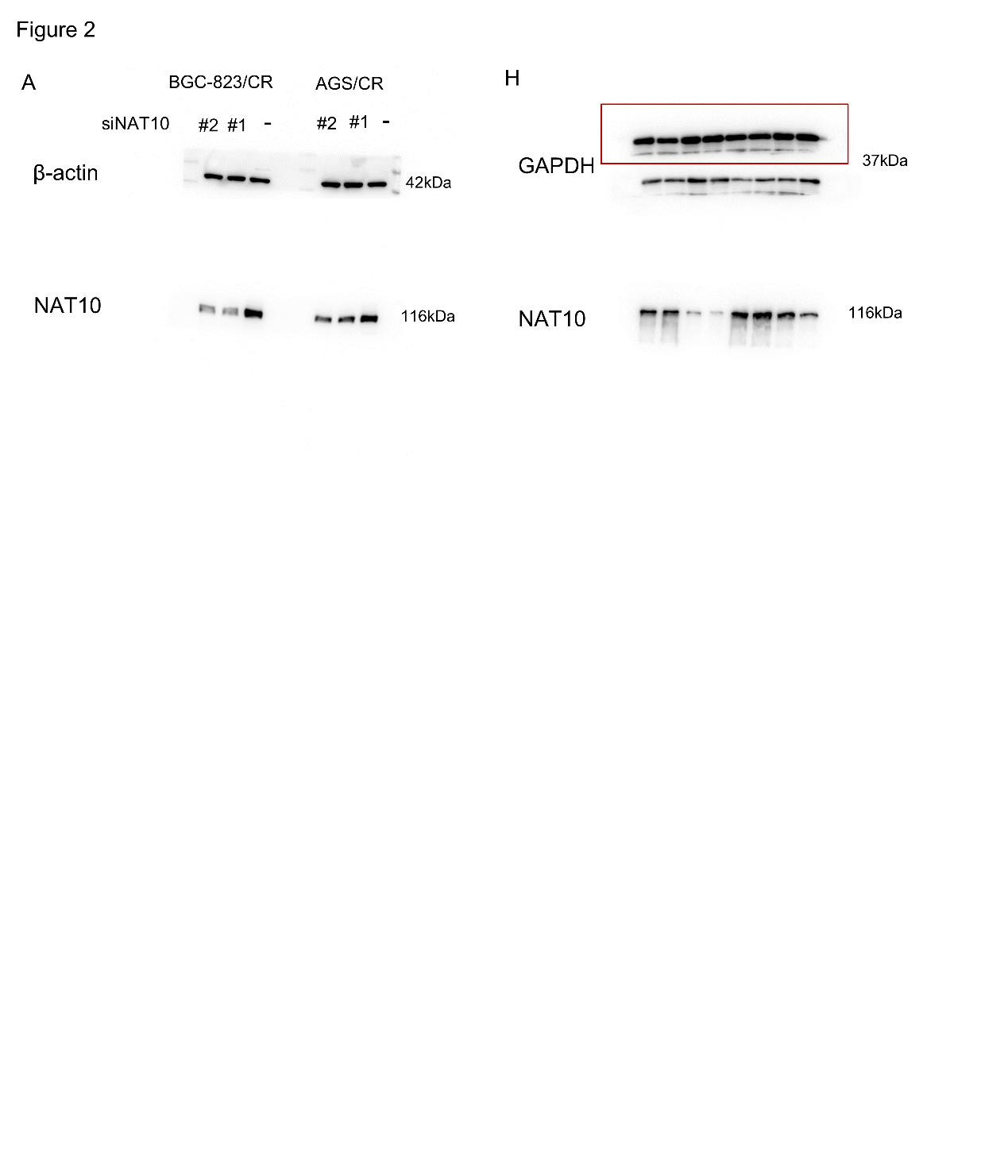

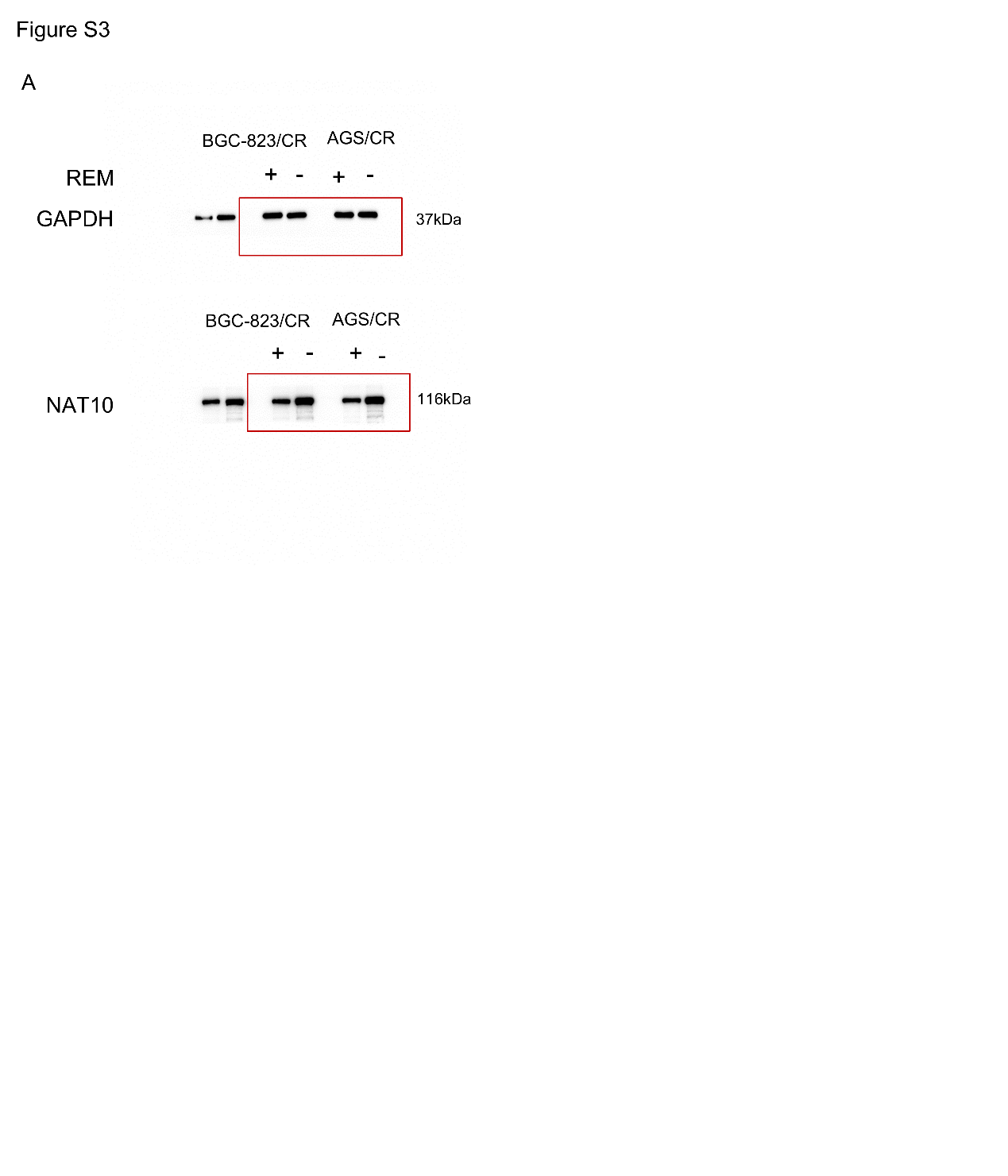

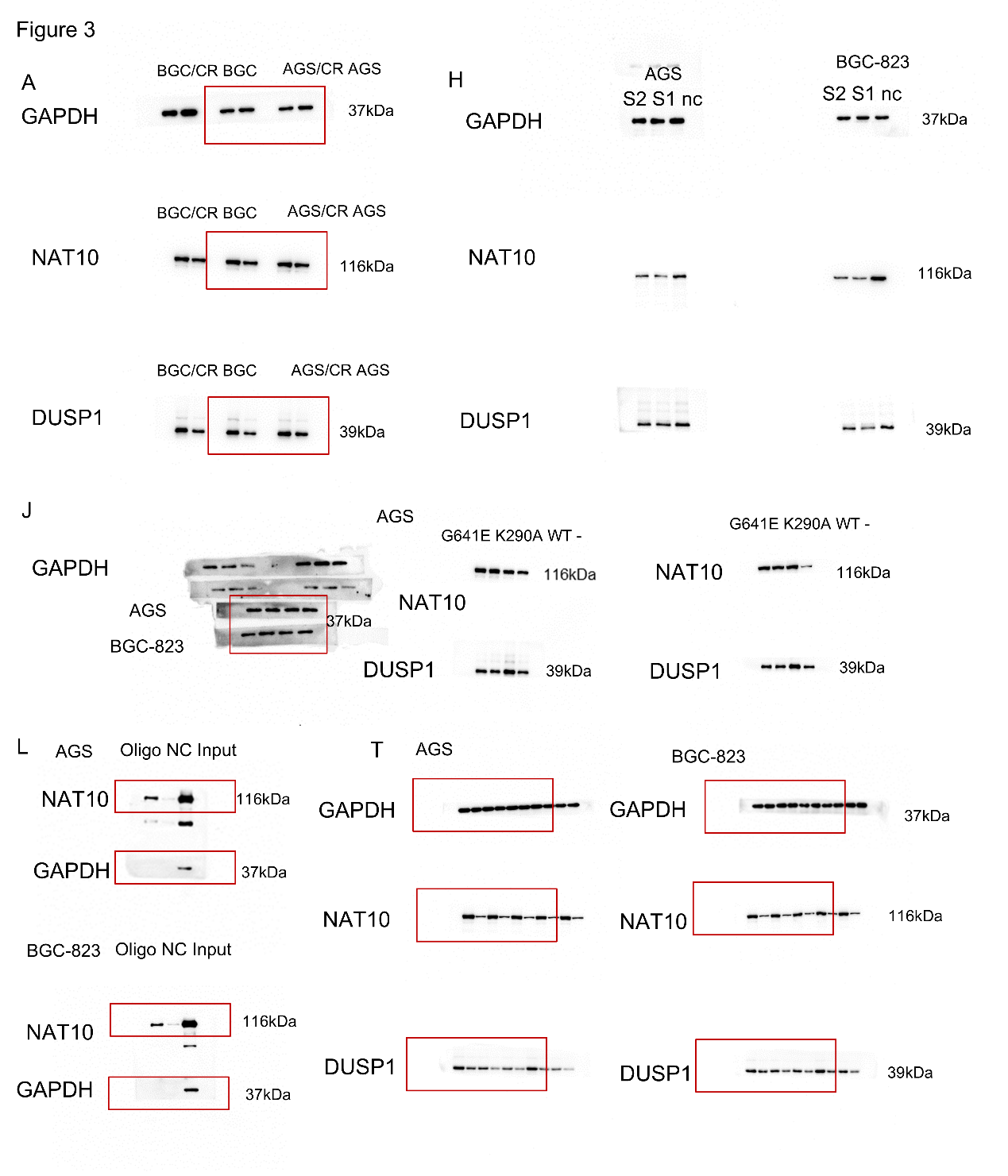

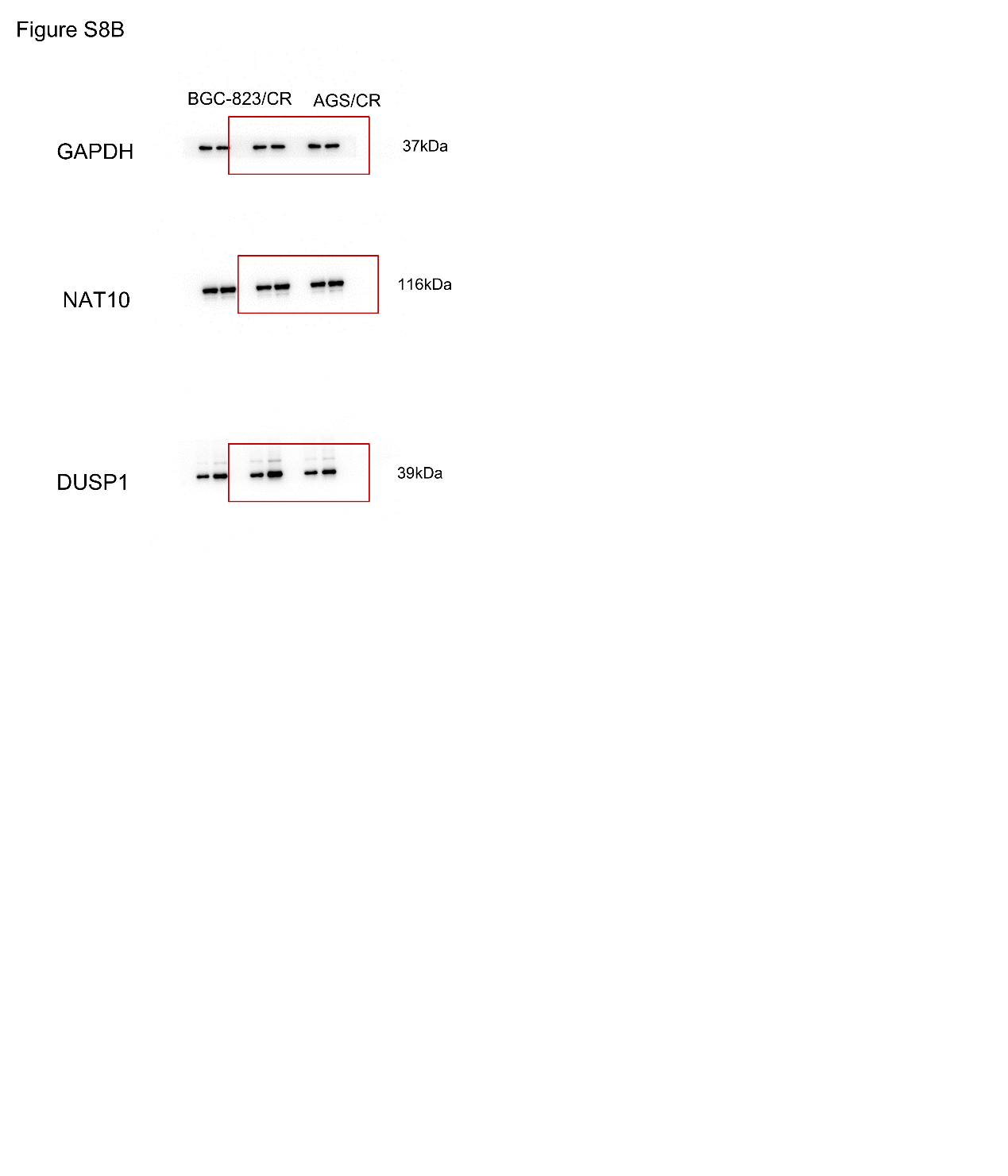

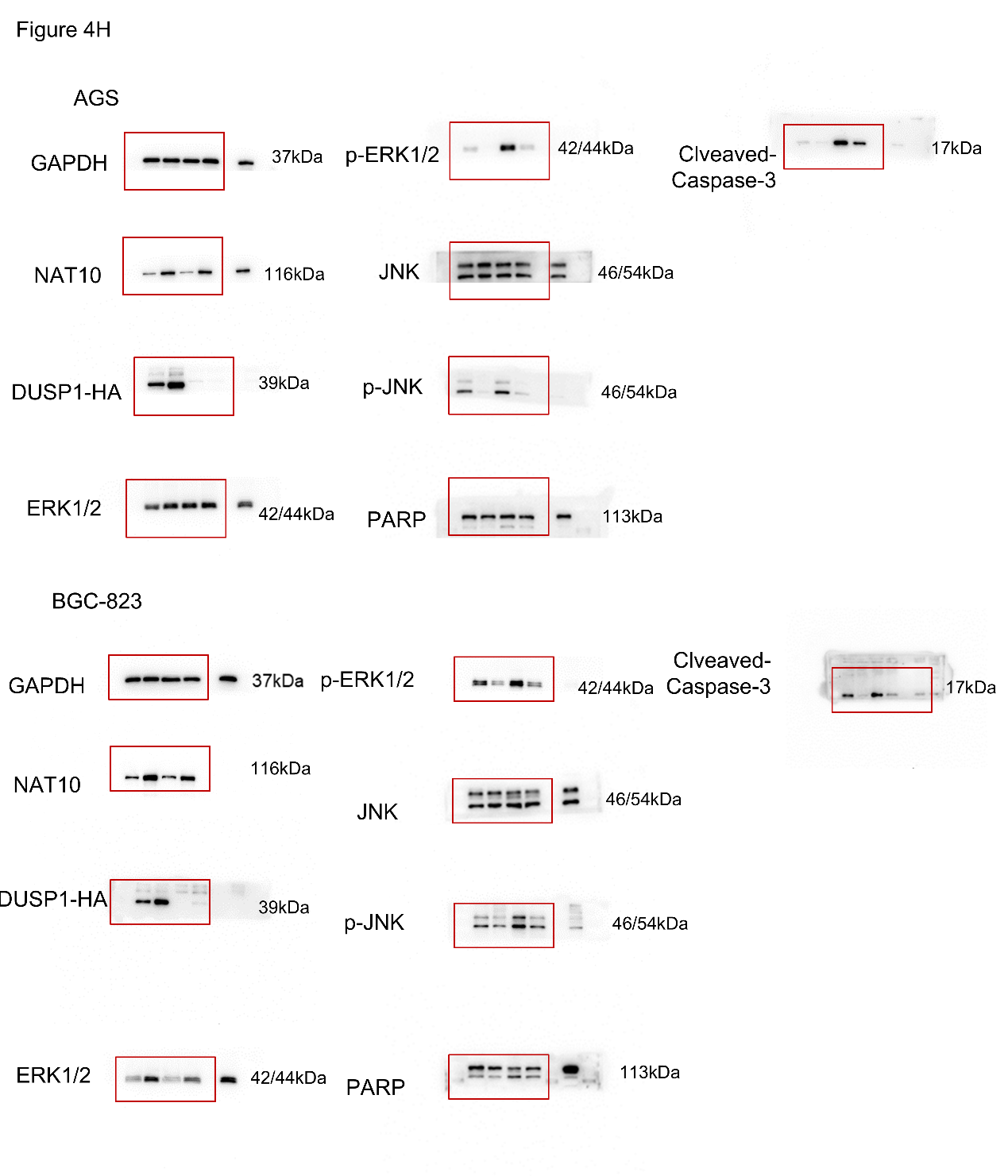

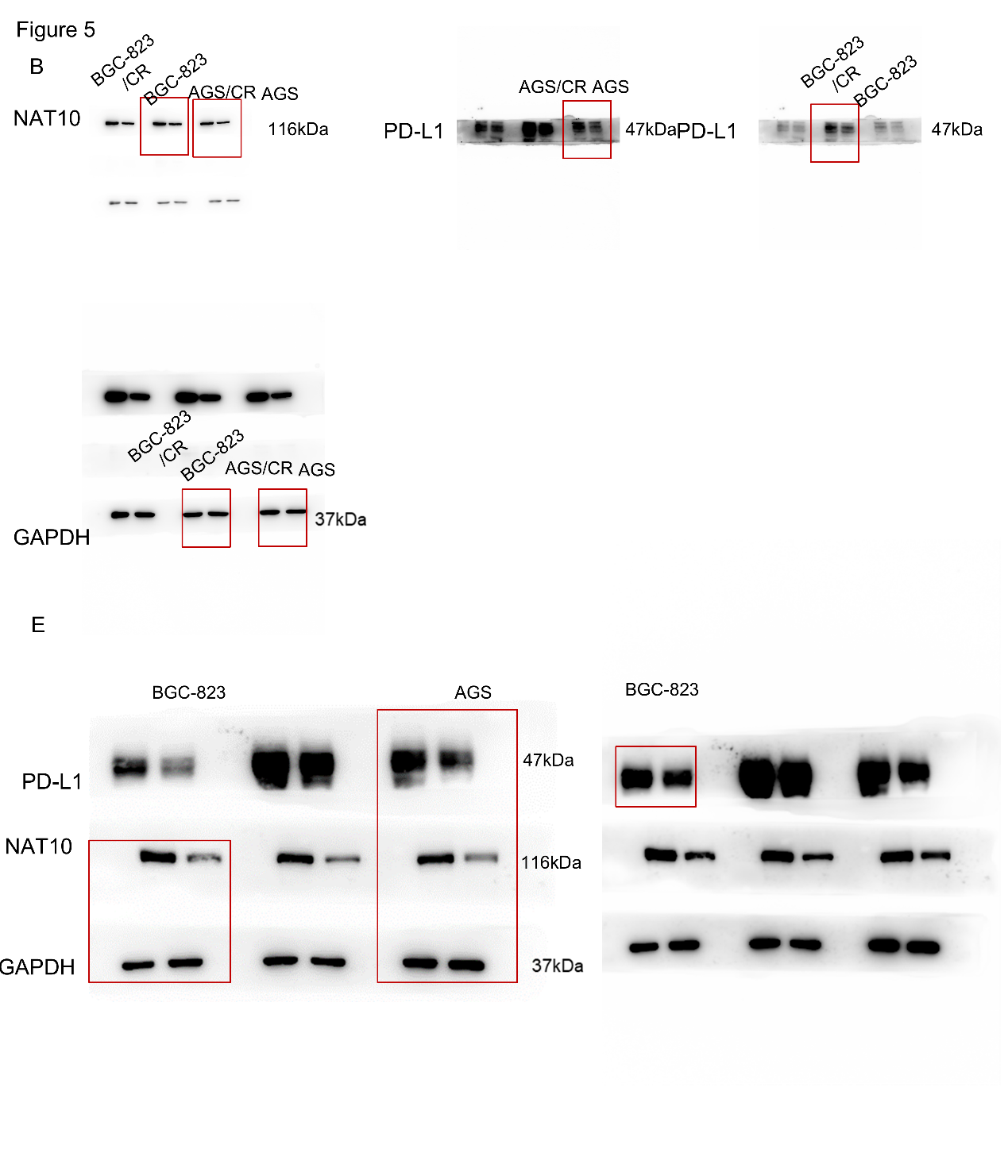

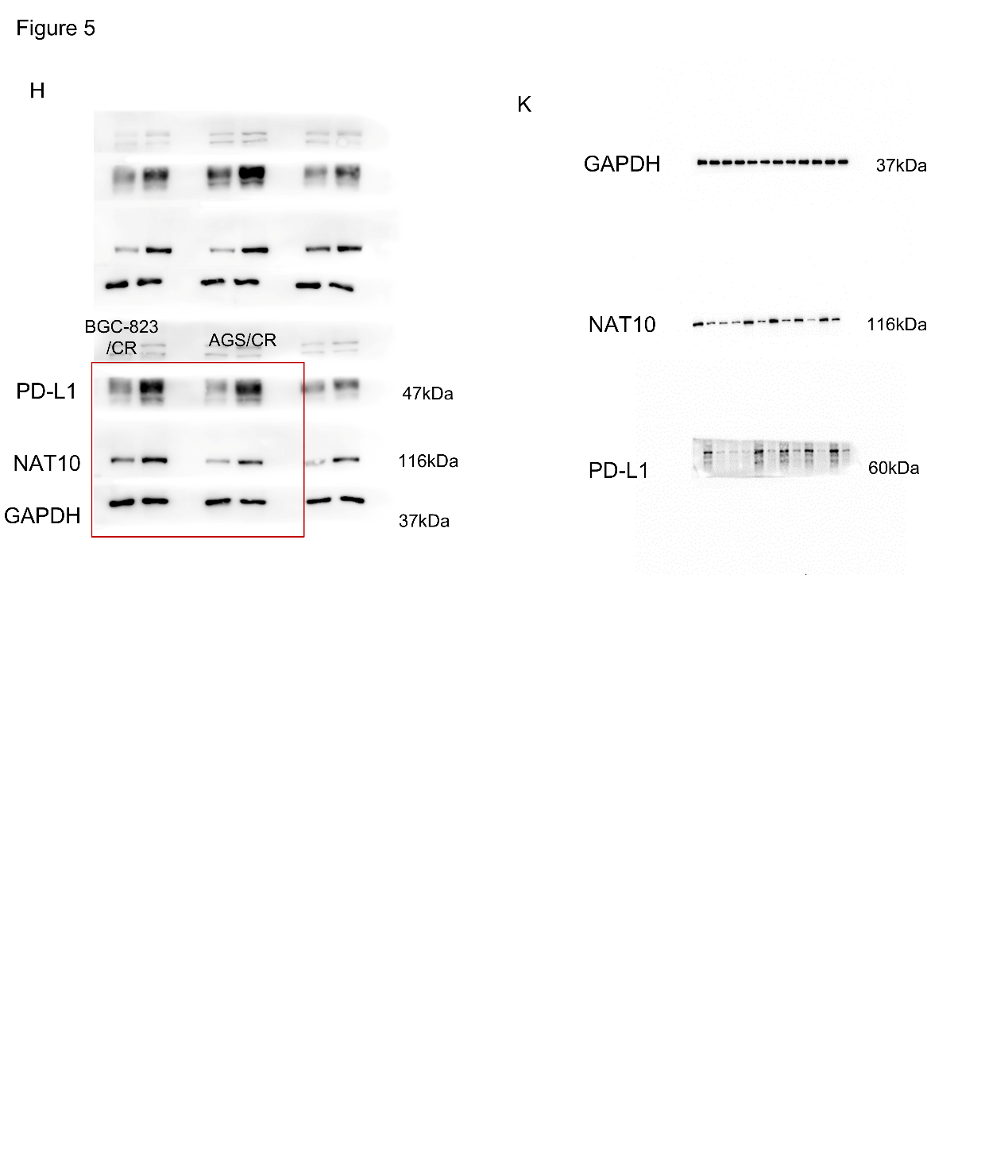

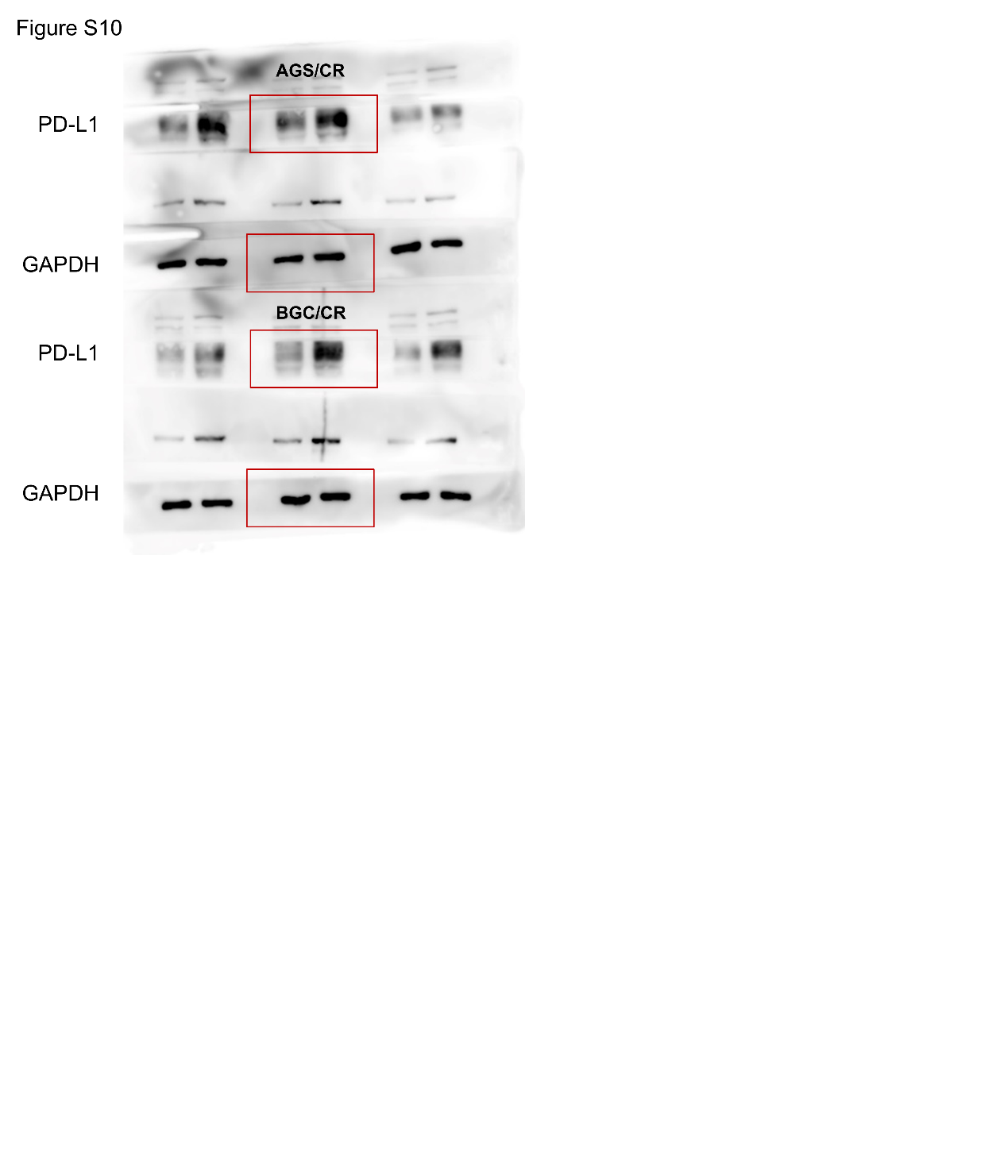

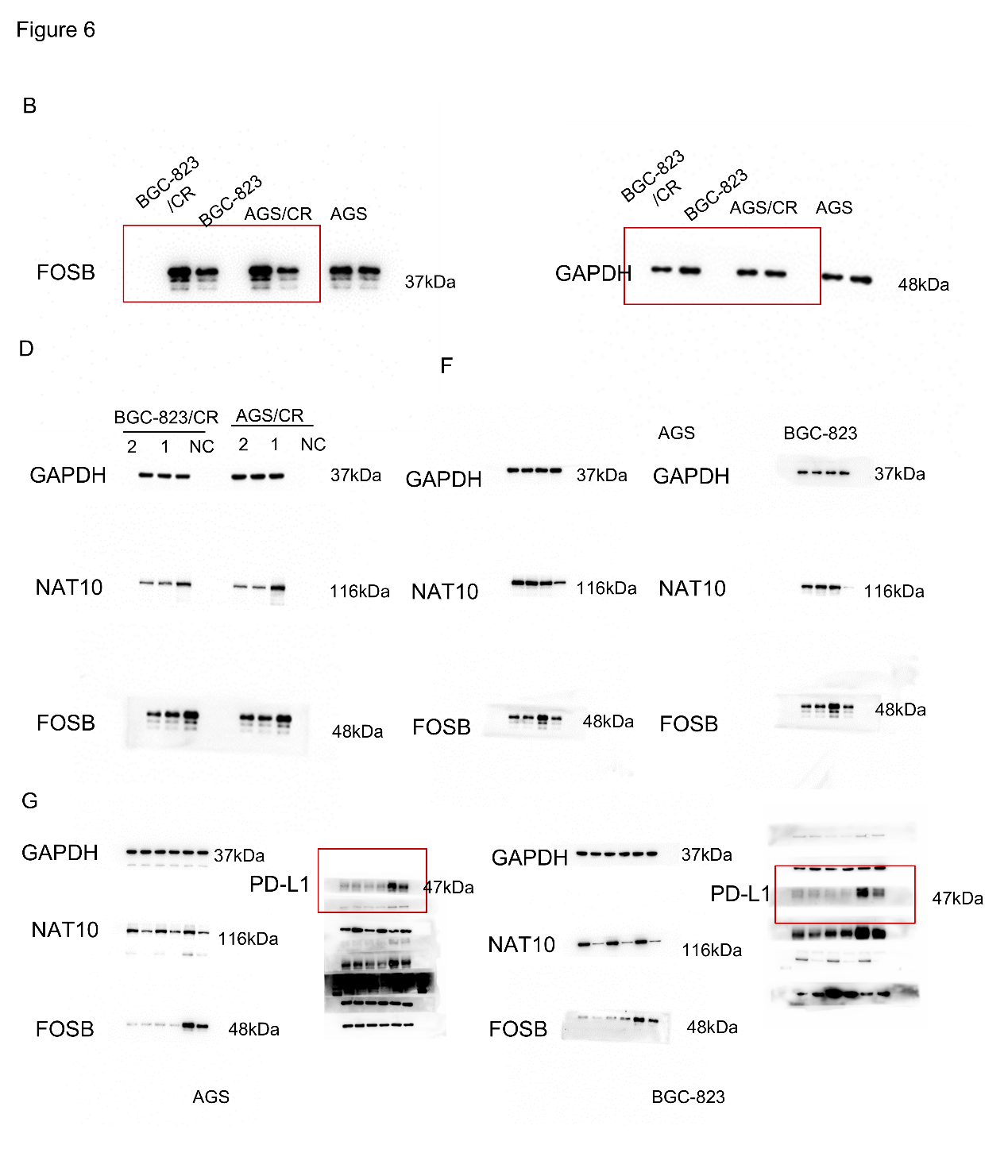

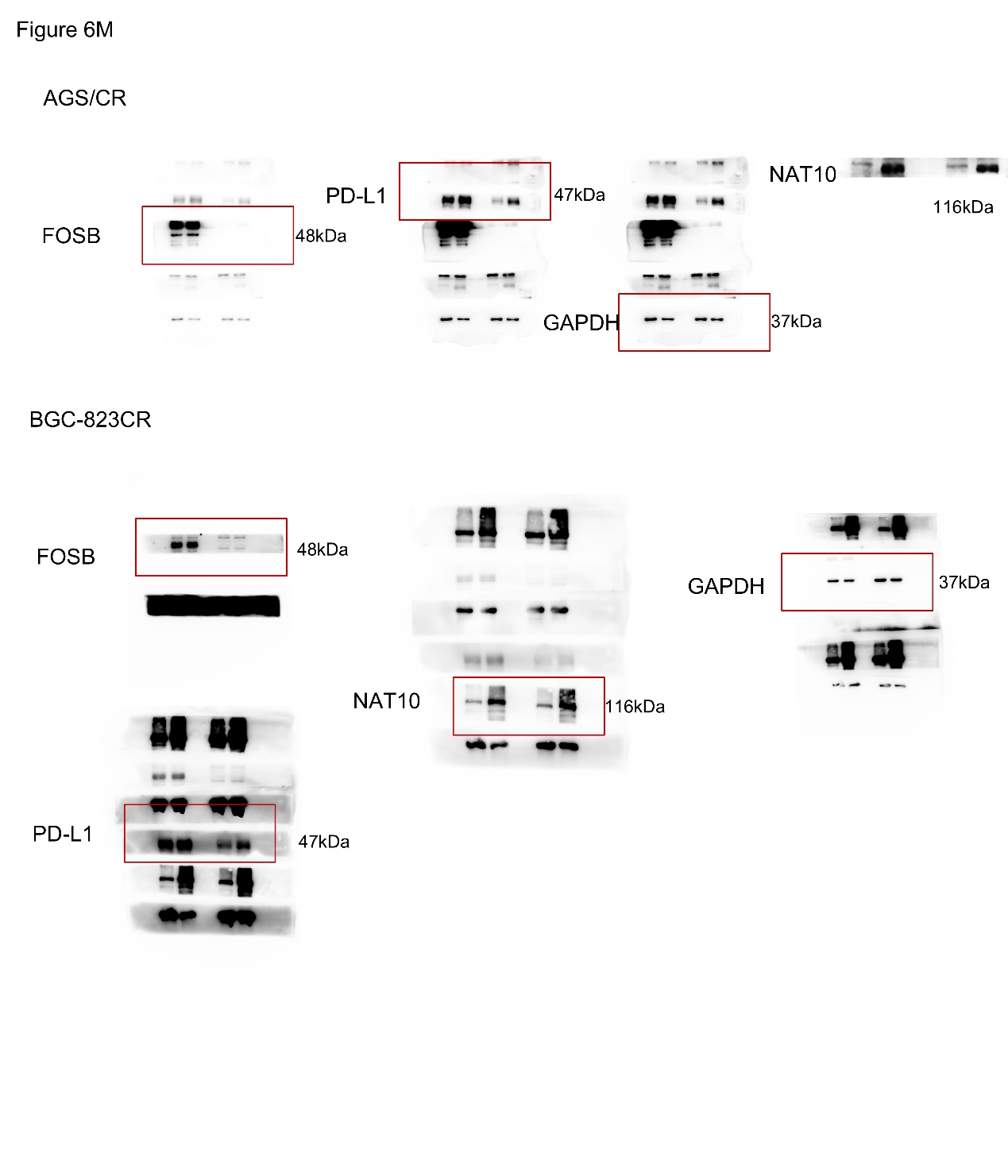

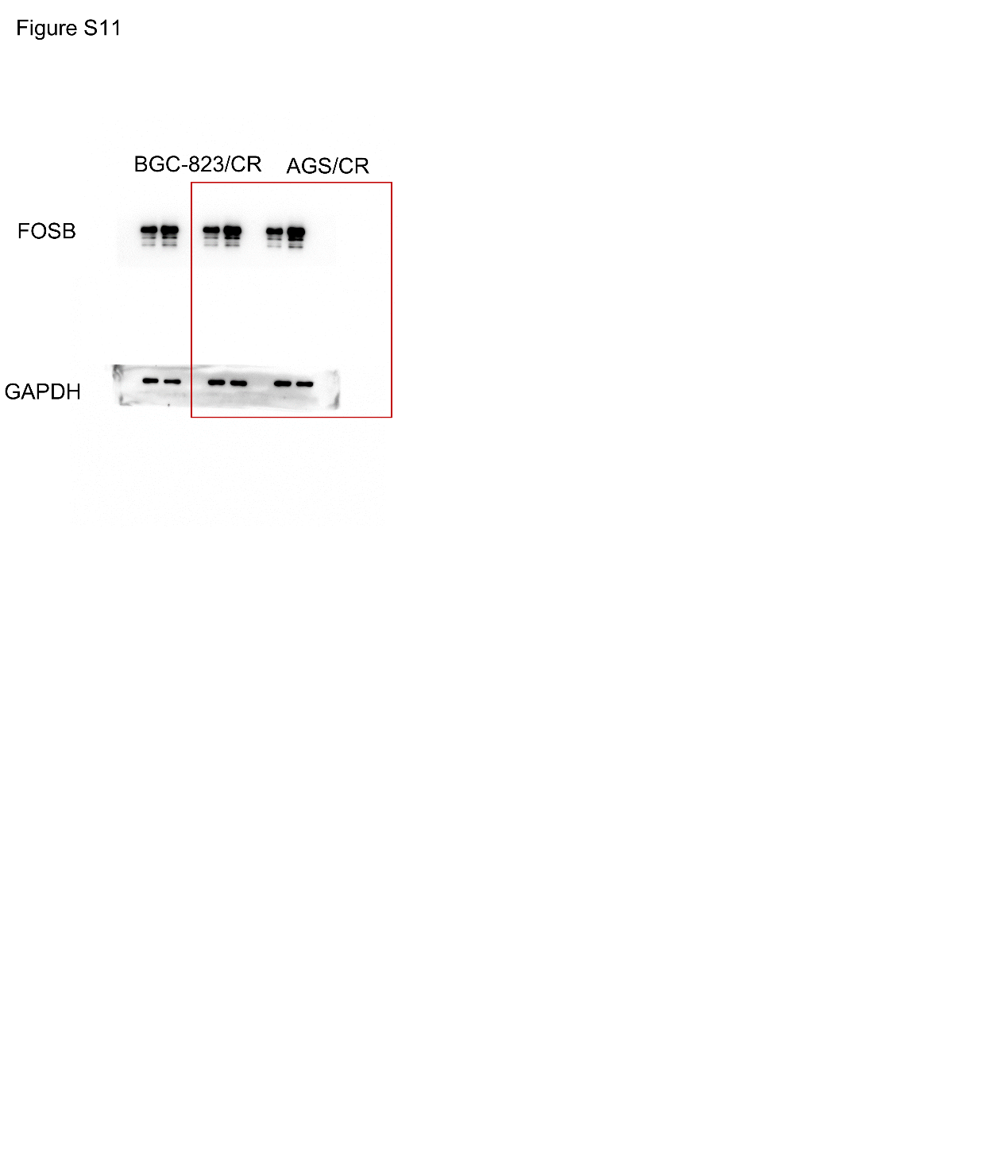

Supplement: Supplementary file 2 — original western blots [file 41420_2026_3107_MOESM2_ESM.docx]
